# Complete Mitochondrial Genomes of Chimpanzee- and Gibbon-Derived *Ascaris* Isolated from a Zoological Garden in Southwest China

**DOI:** 10.1371/journal.pone.0082795

**Published:** 2013-12-17

**Authors:** Yue Xie, Lili Niu, Bo Zhao, Qiang Wang, Xiang Nong, Lin Chen, Xuan Zhou, Xiaobin Gu, Shuxian Wang, Xuerong Peng, Guangyou Yang

**Affiliations:** 1 Department of Parasitology, College of Veterinary Medicine, Sichuan Agricultural University, Ya’an, Sichuan, China; 2 Veterinary Hospital, Chengdu Zoological Garden, Chengdu, Sichuan, China; 3 Department of Chemistry, College of Life and Basic Science, Sichuan Agricultural University, Ya’an, Sichuan, China; University of the Sunshine Coast, Australia

## Abstract

Roundworms (Ascaridida: Nematoda), one of the most common soil-transmitted helminths (STHs), can cause ascariasis in various hosts worldwide, ranging from wild to domestic animals and humans. Despite the veterinary and health importance of the Ascaridida species, little or no attention has been paid to roundworms infecting wild animals including non-human primates due to the current taxon sampling and survey bias in this order. Importantly, there has been considerable controversy over the years as to whether *Ascaris* species infecting non-human primates are the same as or distinct from *Ascaris lumbricoides* infecting humans. Herein, we first characterized the complete mitochondrial genomes of two representative *Ascaris* isolates derived from two non-human primates, namely, chimpanzees (*Pan troglodytes*) and gibbons (*Hylobates hoolock*), in a zoological garden of southwest China and compared them with those of *A. lumbricoides* and the congeneric *Ascaris suum* as well as other related species in the same order, and then used comparative mitogenomics, genome-wide nucleotide sequence identity analysis, and phylogeny to determine whether the parasites from chimpanzees and gibbons represent a single species and share genetic similarity with *A. lumbricoides*. Taken together, our results yielded strong statistical support for the hypothesis that the chimpanzee- and gibbon-derived *Ascaris* represent a single species that is genetically similar to *A. lumbricoides*, consistent with the results of previous morphological and molecular studies. Our finding should enhance public alertness to roundworms originating from chimpanzees and gibbons and the mtDNA data presented here also serves to enrich the resource of markers that can be used in molecular diagnostic, systematic, population genetic, and evolutionary biological studies of parasitic nematodes from either wild or domestic hosts.

## Introduction

Typical metazoan mitochondrial DNA (mtDNA) is a small (14–20 kb), circular, haploid DNA molecule that encodes 12–13 protein-coding genes (PCGs), two ribosomal RNA (rRNA) genes, and 22 transfer RNA (tRNA) genes [1]. Generally, the gene content, order, and organization of mtDNAs are well-conserved across the diverse metazoan groups [2]. This, together with other idiosyncratic characteristics such as faster evolutionary rates than nuclear genes, presumed maternal inheritance, and absence of recombination [3], [4] make mtDNAs one of the most popular targets for population or ecological genetics studies and for phylogenetic and evolutionary analyses [2], [3], [5]. In addition, compared with the conventional individual gene or region, the mtDNA dataset not only provides sets of genome-level information but also effectively reveals inter/intra-species sequence variability and species specificity, thus aiding in the accurate identification and differentiation of a new or cryptic species, including parasitic nematodes [6], [7].

Roundworms (Ascaridida: Nematoda), one of the most common soil-transmitted helminths (STHs), can cause the socioeconomically important disease ascariasis in a wide range of hosts. Mammals, from marsupials to human and non-human primates, birds, reptiles, and fishes, serve as obligatory hosts [8], [9]. Despite the economic and health importance of Ascaridida, the current taxonomic sampling and surveys for Ascaridida are unevenly biased toward the parasitic nematodes that are of great medical or veterinary impact, including *Ascaris lumbricoides*, *Ascaris suum*, *Anisakis simplex*, and *Toxocara canis* (for details see [3], [10]). Little or no attention is paid to roundworms infecting wild animals including non-human primates. It is noteworthy that prior studies have shown that non-human primates living in natural habitats, such as chimpanzees [11], baboons [12], and gibbons [13], can become infected with roundworms that infect humans, indicating that the ascaridoid species harbored in non-human primates could be *A. lumbricoide*s or similar. This conclusion was subsequently supported by several studies based on morphological features [14], [15] and molecular evidence [16], [17]. Nejsum et al. recently demonstrated that chimpanzees can also acquire *A. suum* infections following close association with pigs in captive conditions [18], [19]. Considering the remarkable similarities in morphological characteristics across *Ascaris* spp., including the two well-known species *A. lumbricoides* and *A. suum* in humans and pigs, respectively, and the potential inconsistent phylogenetic estimates based on morphology and molecular markers (e.g., ITS, *cox1*, and *rrn*S), it is still unclear whether *Ascaris* species from non-human primates represent *A. lumbricoides* or *A. suum* or a different species/strain.

With the advent of new technologies associated with PCR, sequencing, and data analysis, increasing attention has been focused on sequencing of the complete mtDNA [10], [20], [21]. Complete mtDNA data can provide novel insights into the genome-wide comparative, phylogenetic-based analyses of Ascaridida. To date, the mtDNAs of 16 Ascaridida species have been completely or almost completely sequenced and deposited in NCBI (as of March 15, 2013; available at http://www.ncbi.nlm.nih.gov/genomes/OrganelleResource.cgi?opt=organelle&taxid=119089). Of these, eight belong to Ascarididae; five, to Toxocaridae; two, to Anisakidae; and one belongs to Cucullanidae [22]–[32]. However, there is no complete information available on the mtDNA of *Ascaris* species infecting non-human primates thus far.

Herein, we elucidated the complete mitochondrial genome sequences of two *Ascaris* isolates obtained from chimpanzees (*Pan troglodytes*) and gibbons (*Hylobates hoolock*) from a southwest zoological garden in China and compared these sequences and genome organizations with those of four complete mtDNAs from the congeneric *A. lumbricoides* (human origin, China and Korea isolates), *A. suum* (pig origin, China and USA isolates) [22], [28], [30], and related nematodes in the same order. Based on comparative mitogenomics, we explored the species identity and diversity between these two *Ascaris* isolates from primates and *Ascaris* species from humans or pigs. In addition, with the help of mVISTA and sliding window analysis, mt genes utilized as molecular markers for characterization and species identification in Ascaridida, including *cytb* and *rrn*L [27], were identified and further confirmed. Finally, we reassessed the phylogenetic relationships of the two *Ascaris* isolates with *A. lumbricoides* and *A. suum* and of the genus *Ascaris* within the order Ascaridida by constructing phylogenetic trees (neighbor-joining [NJ], maximum parsimony [MP], and Bayesian inference [BI]) using both amino acid and nucleotide sequence datasets.

## Materials and Methods

### Ethics statement

This study was reviewed and approved by the Animal Ethics Committee of Sichuan Agricultural University (Ya’an, China; Approval No. 2011–028). The two primates from which adult *Ascaris* specimens were obtained were handled in strict accordance with good animal practice as defined by the Animal Protection Law of the People’s Republic of China (released on September 18, 2009). Details of animal welfare and steps taken to ameliorate suffering were in compliance with the recommendations of the Weatherall report, “The use of non-human primates in research”. Animals were socially caged under an unconstrained and climate-controlled condition (ambient temperature of 21–25°C, humidity of 40–60%, and a 12-h light/dark cycle) when possible or individually caged if no compatible pairing could be found, but rich visual, olfactory, and auditory interactions were permitted during this procedure. Each cage housed two rooms: one indoors and another outdoors. The animals were fed a balanced nutrient diet and fresh fruit twice daily, with water freely available at all times. Moreover, environmental enrichment in the form of ropes, ladders, hanging toys, perches, and other substrates (e.g., rockworks) was provided in the cages to encourage diverse motor activities. The animals received regular veterinary care and monitoring, and all animals survived in good health.

### Parasites and DNA isolation

Adult female specimens of *Ascaris* were obtained from naturally infected chimpanzees and gibbons provided by the Veterinary Hospital, Chengdu Zoological Garden, Sichuan Province of China, after treatment with pyrantel pamoate. Adult worms from each host were washed separately in physiological saline, morphologically identified to the genus level [33], [34], fixed in 70% (v/v) ethanol, and stored at –20°C until use. The total genomic DNA samples from two individual worms (designated cA and gA for the *Ascaris* isolates obtained from the chimpanzee and gibbon, respectively) were extracted using Universal Genomic DNA Extraction Kit Ver. 3.0 (TaKaRa Biotech, Dalian, China). The identity of the two specimens was further verified by PCR amplification of the region spanning the ITS-1, 5.8S, and ITS-2 genes (the ITS region) coupled with the *cox1* gene, and by comparison of the amplified sequences with the previously reported corresponding sequences for the cA (GenBank accession numbers JF837180 and EU628688) and gA (GenBank accession numbers JF837181 and EU628687) [17]. Both the ITS and *cox1* sequences from either cA or gA determined here shared ≥ 99.5% similarities to those mentioned above.

### PCR amplification and sequencing

Primers (**Table S1**) were designed on the basis of the alignments of the relatively conserved regions of congeneric *A. lumbricoides* and *A. suum*, and *Baylisascaris procyonis* mtDNA sequences, and the entire mt genome of each specimen was amplified in 12 overlapping segments (ranging from 875 bp to 2.2 kb in size) via PCR. All PCR reactions were conducted in a final reaction volume of 25 µL containing 15–20 ng of genomic DNA, 1 U of Ex *Taq* Polymerase (TaKaRa), 10× Ex *Taq* buffer (Mg^2+^ Plus), 2.5 mM of dNTP mixture, 10 pmol of each primer, and ddH_2_O. The reaction was carried out in a Mastercycler Gradient 5331 thermocycler (Ependorf, Germany) at 94°C for 5 min (initial denaturation); followed by 35 cycles of 94°C for 30 s (denaturation), 50–55°C for 30 s (annealing), and 68°C for 2–6 min (extension) according to the product length; with a final extension step at 68°C for 10 min. Each PCR yielded a single band, which could be visualized after electrophoresis in 1% (w/v) agarose gel and ethidium bromide staining **(data not shown)**. Each amplicon was then column-purified using the AxyPrep DNA Gel Extraction Kit (Axygen, Hangzhou, China) and subjected to automated sequencing, either directly or after it was subcloned into the pMD19-T vector (TaKaRa), using a ‘‘primer-walking’’ strategy. Bidirectional sequencing was performed using terminator-based cycle sequencing with BigDye chemistry (Applied Biosystems, Foster City, CA) on an ABI-3730 DNA sequencer (Applied Biosystems). To ensure maximum accuracy, each amplicon was sequenced twice independently, and a third PCR product was sequenced in case of discrepancies.

### Genome assembly, annotation, and sequence variation analysis

The consensus sequences were manually assembled into a single contig and aligned against the published complete mtDNA sequences of *A. lumbricoides* and *A. suum* [22], [28], [30] using ClustalX version 1.83 [35], and the circular map was drawn using the program MacVector version 9.5 (http://www.macvector. com/index.html). Annotation, genome-scale “synteny” (usually described in plant or fungus mtDNAs) and comparisons with *A. lumbricoides* and *A. suum* and other nematodes in the same order were performed using an automatic organelle genome annotation program, DOGMA [36], combined with DNAMAN version 3.0 (Lynnon Biosoft, Quebec, Canada) and online BLAST search tools available through the NCBI website [37]. The base composition and codon usage were calculated with MEGA 4.0 [38]. Secondary structures of tRNAs, rRNAs, and non-coding regions (NCRs) were inferred using standard approaches [39]. The complete nucleotide sequences of the mtDNAs obtained from 17 Ascaridida species (including those from chimpanzees and gibbons) were separately aligned into different genera (*Ascaris*, *Baylisascaris*, *Toxocara*, *Anisakis*, *Contracaecum*, and *Cucullanus*) using MEGA software, and the complete alignments were subsequently used to carry out sliding window analyses with DnaSP version 5.10 [40]. A sliding window of 300 bp with 30-bp steps was used to estimate the nucleotide diversity Pi (π) for these alignments. Nucleotide diversity for the complete alignment was plotted against midpoint positions of each window, and gene boundaries were indicated. Additionally, DnaSP was used to calculate the number of synonymous substitutions per synonymous site (Ks) and the number of nonsynonymous substitutions per nonsynonymous site (Ka) for each PCG in Ascaridida, but the stop codons were not taken into the analysis because of the presence of an incomplete termination codon (T). Based on π values, together with the results of the mVISTA [41] genome comparison analysis and nucleotide and/or amino acid sequence divergence within and between mt genes among the available Ascaridida mtDNAs, the regions/genes that could be aligned among the 17 genomes and showed sequence divergence were extracted for marker identification. To obtain clear informative character, these regions/genes were realigned using ClustalX with manual adjustment. Of these, more reliable or more informative fragments were screened and evaluated for their applicability as molecular markers for species characterization and identification using PCR. The corresponding PCR primer pairs for amplification were designed as previously described by Xie et al. (2011) [27]. All PCR reactions contained approximately 20 ng of the genomic DNA extracted from cA, gA, *A. lumbricoides*, *A. suum*, *B. procyonis*, *Baylisascaris schroederi*, *Baylisascaris ailuri*, *Baylisascaris transfuga*, *T. canis*, and *Toxocara leonine* (provided by Sichuan Agriculture University, China) and were carried out in a 25-µL reaction mixture with 2 mM MgCl_2_, 2.5 mM of each dNTP, 50 µM of 10× PCR buffer (Mg^2+^ Free), 1 U *Taq* DNA polymerase (TaKaRa), 10 pmol of each primer, and ddH_2_O under the following cycle conditions: an initial denaturation at 95°C for 3 min; 30 cycles at 95°C for 30 s, 48–52°C for 30 s, and 72°C for 40 s; and a final step at 72°C for 8 min.

### Phylogenetic analyses

Amino acid sequences inferred from 12 PCGs and the respective nucleotide sequences for 12 PCGs, 2 rRNAs plus 22 tRNAs, and all PCGs and RNAs together, common among all of the nematodes investigated here, were separately concatenated into four single alignments (one amino acid alignment and three nucleotide alignments), and aligned against those of 17 other chromadorean nematodes using both T-Coffee 7.81 [42] and ClustalX with default parameters for subsequent phylogeny. The nucleotide alignments involved in the PCGs were generated on the basis of the protein alignment using codon alignment. The nematodes used included: *A. lumbricoides* (China isolate; GenBank: HQ704900), *A. lumbricoides* (Korea isolate; GenBank: JN801161), *A. suum* (China isolate; GenBank: HQ704901), *A. suum* (USA isolate; GenBank: NC_001327), *B. procyonis* (GenBank: JF951366), *B. ailuri* (GenBank: HQ671080), *B. schroederi* (GenBank: HQ671081), *B. transfuga* (GenBank: HQ671079), *T. canis* (Australia isolate; GenBank: EU730761), *T. canis* (China isolate; GenBank: NC_010690), *Toxocara cati* (GenBank: NC_010773), *Toxocara malaysiensis* (GenBank: NC_010527), *A. simplex* (GenBank: NC_007934), *Contracaecum rudolphii* B (GenBank: NC_014870), *Cucullanus robustus* (GenBank: NC_016128), *Dirofilaria immitis* (GenBank: NC_005305), and *Onchocerca volvulus* (GenBank: NC_001861). During the procedure, any ambiguous regions within these alignments were filtered with Gblocks 0.91 b. [43]. Phylogenetic analyses were performed with three methods: NJ, MP, and BI, using the 17 Ascaridida isolates (cA, gA, *A. lumbricoides* (two isolates), *A. suum* (two isolates), *B. ailuri*, *B. procyonis*, *B. schroederi*, *B. transfuga*, *T. canis* (two isolates), *T. cati*, *T. malaysiensis*, *A. simplex*, *C. rudolphii* B, and *C. robustus*) as ingroups and two Filarioidea species (*D. immitis* and *O. volvulus*) as outgroups in each analysis. In the first concatenated amino acid sequence dataset, NJ and MP analyses were conducted as described previously [27]. For BI, phylogenetic reconstructions were carried out using MrBayes version 3.1.2 (http://mrbayes.csit.fsu.edu/index.php) [44] with four independent Markov chain runs for 1,000,000 metropolis-coupled Monte Carlo (MCMC) generations, sampling a tree every 1,000 generations. When the average standard deviation of the split frequencies had reduced to less than 0.01, the first 250 trees (25%) were discarded as “burn-in” and the remaining trees were used to calculate Bayesian posterior probabilities. Evolutionary distance was estimated using the MrBayes order (aamodelpr  =  mixed), allowing for a gamma-shaped variation in mutation rates with a proportion of invariable sites (rates  =  invgamma). A consensus tree was obtained and constructed in TreeviewX (http://darwin.zoology.gla.ac.uk/rpage/treeviewx/). In the second nucleotide sequence dataset, data for the PCGs and rRNAs plus tRNAs were analyzed separately and in combination (i.e., all 12 PCGs, both RNAs (2 rRNAs + 22 tRNAs), and then all PCGs and RNAs together) using the MP and BI methods and the same criteria employed for amino acid sequence data analyses. For NJ, the GTR+I+G model determined by ModelTest 3.7 [45] using Akaike’s information criterion (AIC) was optimum and was applied in the following NJ analysis.

## Results and Discussion

### Genome structure and organization

**Figure 1:**
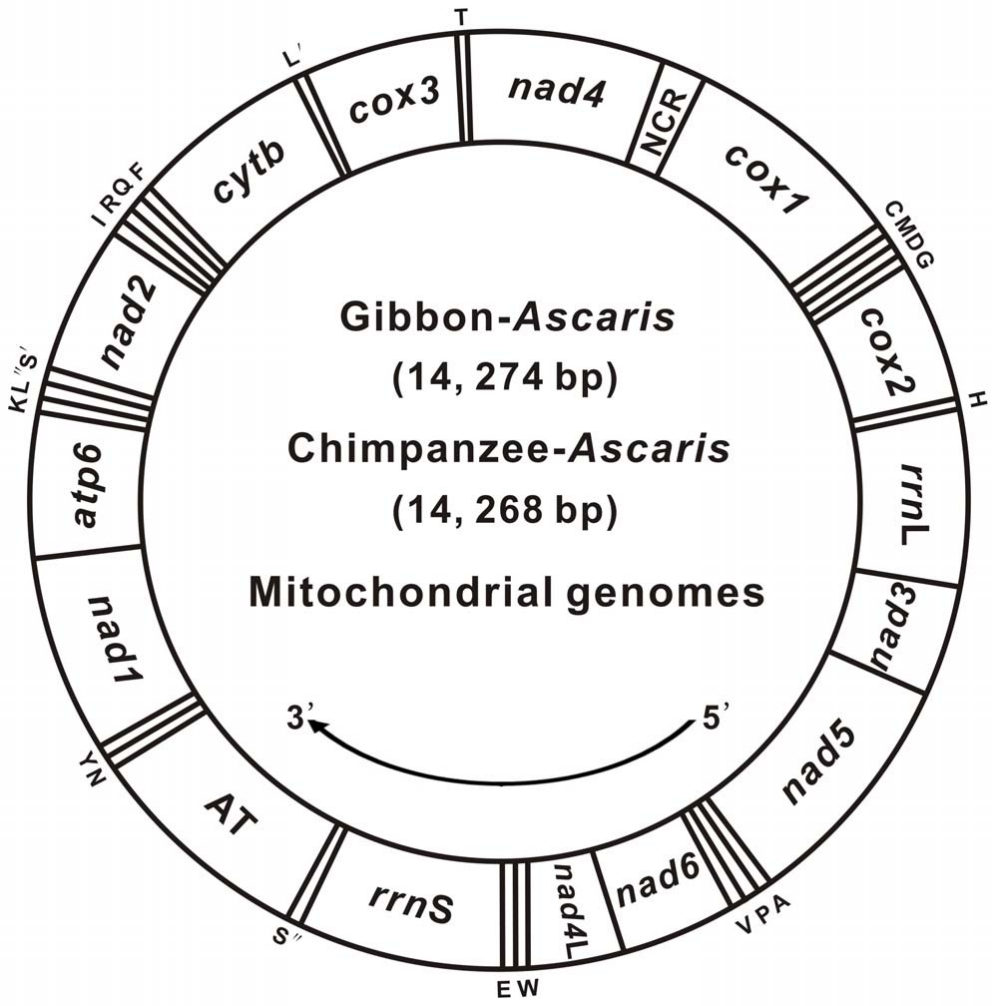
Circular mitochondrial genome map for *Ascaris* obtained from chimpanzees and gibbons. Genes follow standard nomenclature [27], including the 22 tRNA genes, which are indicated by one letter according to the IPUC-IUB single-letter amino acid codes. The two leucine genes were differentiated by L′ (CUN) and L″ (UUR), and the two serine genes by S′ (AGN) and S″ (UCN). All the genes are located on the same strand and transcribed in the same direction (5′–3′), as indicated by the arrow. AT refers to the AT-rich region; NCR refers to the non-coding region.

The circular mt genome sequences for cA and gA were 14,268 bp and 14,274 bp in size, respectively (GenBank: KC839986 and KC839987, respectively; **Figure 1**), well within the genome size range (13,916–14,898 bp) of other Ascaridida nematodes [46]. As with most nematode mtDNAs (except *Trichinella spiralis*), both genomes encoded the entire set of 36 genes, including 12 PCGs (*atp6*, *cox1–3*, *cytb*, *nad1–6*, and *nad4*L), 22 transfer RNA (*tRNA*) genes, and 2 ribosomal RNAs (*rrn*S and *rrn*L), but lacked the *atp*8 gene (**Figure 1** **; Table S2**). All the genes were located on the same strand and transcribed in the same direction (5′ to 3′), typical for all secernentean nematodes characterized to date (**Figure 1**). Only one long unassigned region was found between the *rrn*S and *nad1* genes, flanked at the 5′-end by the *tRNA*-Ser^UCN^ gene and at the 3′-end by the *tRNA*-Asn and *tRNA*-Tyr genes, and this region was deemed homologous to the AT-rich region by analysis of positional homology, potential structure, and nucleotide composition. In addition, one overlap involving one base pair was present at the junction between *cox1* and *tRNA*-Cys. Across both genomes, 81 nucleotides were found scattered in 13 intergenic spacers ranging from 1–16 bp in size excluding the NCR. Of these, two spacers were >10 bp in length, with one 14-bp spacer present between the *cytb* and *tRNA*-Leu^CUN^ genes and another 16-bp spacer between the *rrn*S and *tRNA*-Ser^UCN^ genes (see **Table S2**).

The gene orders of cA and gA mtDNAs followed the gene arrangement (GA7) [47] and were similar with those of the other Ascaridida and Strongylida members and the free-living nematode *Caenorhabditis elegans*, with the exception of the relative positions of the AT-rich region and the number of NCRs [22]–[30], [32], [39], [48]. Further synteny analysis in Ascaridida revealed that the mt genomes of both *Ascaris* isolates studied here featured maximum (100%) synteny with representatives from the genera *Baylisascaris*, *Toxocara*, *Anisakis*, and *Contracaecum*, as well as with the congeneric species *A. lumbricoides* and a comparable synteny with *C. robustus* within the genus *Cucullanus* (**Figure S1**). However, the mt genomes of both species failed to show such levels of synteny with *D. immitis* and *O. volvulus* genomes (order Spirurida) and with those of any other chromadorean nematodes described to date. In general, the conserved synteny groups/units (comprised of regions or genes) are shared between the species only within the same order and, to a certain extent, this implies a common ancestry or evolutionary route [49], [50]. Such high synteny combined with the consistent gene order, including the AT-rich region and NCRs, was observed between the two *Ascaris* isolates and Ascaridida species mtDNAs, indicating that cA and gA are more closely related to Ascaridida species than to other secernentean nematodes.

### Nucleotide composition and codon usage

As expected for other nematodes, the nucleotide usage (coding strand) in the entire mtDNA sequences for cA and gA were biased toward A and T (both mtDNAs: A  =  22.1%, T  =  49.7%, G  =  20.4%, and C  =  7.8%; **Table 1**). The total A + T contents were 70.3% and 74.3% for the PCGs and rRNAs, respectively, and those were 70.9% and 71.0% for the tRNAs, 75.9% and 76.1% for the NCR, and 84.7% and 84.6% for the AT-rich region in the case of cA and gA, respectively, together yielding an aggregate A + T composition of 71.8% in each mtDNA. This value was identical to the A + T content reported in *A. lumbricoides* (Korea isolate) and *A. suum* (China isolate) and was comparable to that in *A. lumbricoides* (China isolate) and *A. suum* (USA isolate), thus making cA and gA mtDNAs one of the genomes with the highest AT content in this order (**Tables 1** **and S3**). This strand base usage bias (strand asymmetry) is usually called “skewness” [51], which can be measured as (A% – T%)/(A% + T%) and (G% – C%)/(G% + C%). A comparative analysis of AT and GC skews across all the available ascaridoid mtDNAs is shown in **Table 1**. The composition of the complete mtDNA sequences of these two *Ascaris* isolates consistently exhibited an extreme skew away from A or C in favor of T or G, with an AT skew of –0.384 and GC skew of 0.447, which was highly congruent with those observed in the mtDNA sequences of *Ascaris* and *Baylisascaris* **(** **Table 1** **)**. Previous studies have proposed that the strand compositional asymmetry could be attributed to the asymmetrical directional mutation pressure arisen from the mtDNA replication, and recent evidence from the investigations on the spontaneous deamination process of C and A during mammal mtDNA replication appeared to confirm this conclusion [52], [53].

**Table 1:** Summary on the nucleotide composition of the mitochondrial genomes of 17 Ascaridida nematodes.

Family	Species (Location)	Host	Size (bp)	No. of			Nt frequecy (%)				EmtG^b^ sequence			References
				PCGs^a^	rRNAs	tRNAs	A	T	G	C	A+T%	AT skew	GC skew	
**Ascarididae**	***Ascaris lumbricoides*** ** (China)**	**Human**	**14,303**	**12**	**2**	**22**	**22.1**	**49.6**	**20.4**	**7.9**	**71.7**	–**0.383**	**0.442**	**Liu et al. (2012)**
	***Ascaris lumbricoides*** ** (Korea)**	**Human**	**14,281**	**12**	**2**	**22**	**22.1**	**49.7**	**20.4**	**7.8**	**71.8**	–**0.384**	**0.447**	**Park et al. (2011)**
	***Ascaris*** ** sp. (China)**	**Chimpanzee**	**14,268**	**12**	**2**	**22**	**22.1**	**49.7**	**20.4**	**7.8**	**71.8**	–**0.384**	**0.447**	**This study**
	***Ascaris*** ** sp. (China)**	**Gibbon**	**14,274**	**12**	**2**	**22**	**22.1**	**49.7**	**20.4**	**7.8**	**71.8**	–**0.384**	**0.447**	**This study**
	***Ascaris suum*** ** (China)**	**Pig**	**14,311**	**12**	**2**	**22**	**22.1**	**49.7**	**20.5**	**7.7**	**71.8**	–**0.384**	**0.454**	**Liu et al. (2012)**
	***Ascaris suum*** ** (USA)**	**Pig**	**14,284**	**12**	**2**	**22**	**22.1**	**49.8**	**20.4**	**7.7**	**71.9**	–**0.385**	**0.452**	**Okimoto et al. (1992)**
	***Baylisascaris ailuri*** ** (China)**	**Red panda**	**14,657**	**12**	**2**	**22**	**21.6**	**47.9**	**22.1**	**8.4**	**69.5**	–**0.378**	**0.449**	**Xie et al. (2011)**
	***Baylisascaris procyonis*** ** (China)**	**Raccoon**	**14,781**	**12**	**2**	**22**	**22.0**	**48.5**	**21.4**	**8.1**	**70.5**	–**0.376**	**0.448**	**Xie et al. (2011)**
	***Baylisascaris schroederi*** ** (China)**	**Giant panda**	**14,778**	**12**	**2**	**22**	**22.1**	**47.5**	**22.9**	**8.5**	**68.6**	–**0.365**	**0.457**	**Xie et al. (2011)**
	***Baylisascaris transfuga*** ** (China)**	**Bear**	**14,898**	**12**	**2**	**22**	**21.6**	**47.8**	**22.1**	**8.5**	**69.4**	–**0.377**	**0.444**	**Xie et al. (2011)**
**Toxocaridae**	***Toxocara canis*** ** (Australia)**	**Fox**	**14,163**	**12**	**2**	**22**	**21.6**	**46.7**	**22.1**	**9.4**	**68.3**	–**0.367**	**0.403**	**Jex et al. (2008)**
	***Toxocara canis*** ** (China)**	**Dog**	**14,322**	**12**	**2**	**22**	**21.9**	**46.7**	**22.0**	**9.4**	**68.6**	–**0.361**	**0.401**	**Li et al. (2008)**
	***Toxocara cati*** ** (China)**	**Cat**	**14,029**	**12**	**2**	**22**	**22.3**	**47.7**	**20.9**	**9.1**	**70.0**	–**0.363**	**0.393**	**Li et al. (2008)**
	***Toxocara malaysiensis*** ** (China)**	**Cat**	**14,266**	**12**	**2**	**22**	**21.7**	**47.2**	**22.0**	**9.1**	**68.9**	–**0.370**	**0.415**	**Li et al. (2008)**
**Anisakidae**	***Anisakis simplex*** ** (Korea)**	**Fish**	**13,916**	**12**	**2**	**22**	**22.9**	**48.3**	**19.0**	**9.8**	**71.2**	–**0.357**	**0.320**	**Kim et al. (2006)**
	***Contracaecum rudolphii*** ** B (China)**	**Cormorant**	**14,022**	**12**	**2**	**22**	**22.8**	**47.6**	**19.7**	**9.9**	**70.4**	–**0.353**	**0.331**	**Lin et al. (2012)**
**Cucullanidae**	***Cucullanus robustus*** ** (Korea)**	**Eel**	**13,972**	**12**	**2**	**22**	**24.6**	**47.0**	**19.3**	**9.1**	**71.6**	–**0.313**	**0.359**	**Park et al. (2011)**

^a^ Protein-coding genes.

^b^ Entire mitochondrial genome.

**Figure 2:**
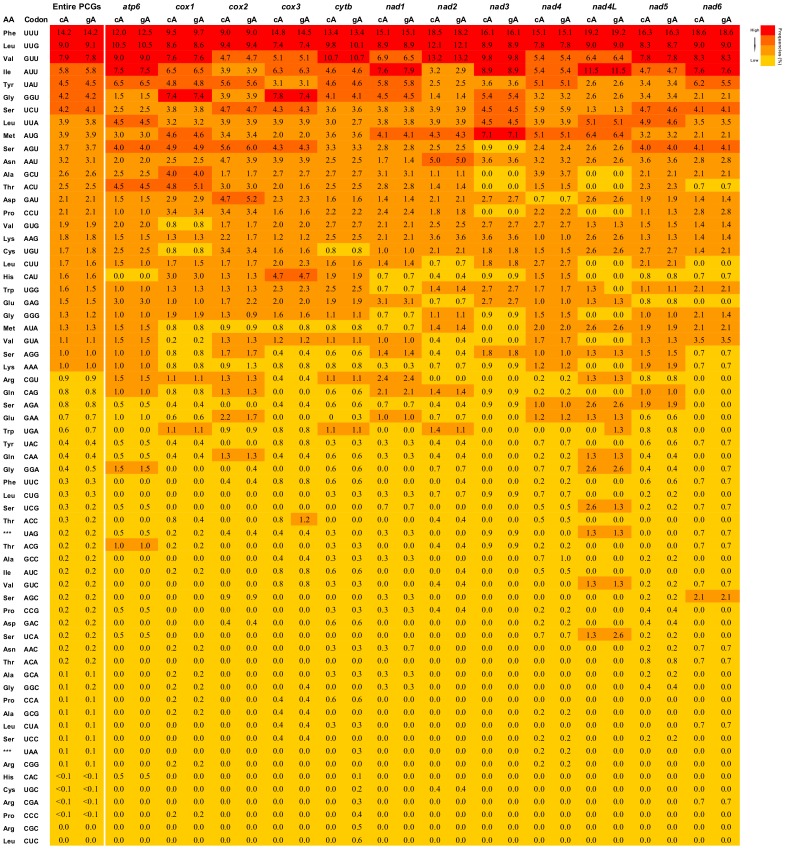
Frequency profile of codons in PCGs of chimpanzee *Ascaris* (cA) and gibbon *Ascaris* (gA) mtDNAs. Colors from red (high frequency) to yellow (low frequency) indicate the different frequencies of codon usage.

Additionally, the genome-wide nucleotide biases were well also reflected in codon usage patterns of PCGs. In cA and gA mtDNAs, a considerable codon usage bias was identified in PCGs (**Figure 2** **)**. Overall, UUU (Phe), UUG (Leu), GUU (Val), and AUU (Ile) were the dominant codons, consistent with data of other nematode species [22]–[30], [32], [39], [48]. However, the proportion of these codons showed an evident variation in different PCGs. For instance, UUU (Phe) was present at over 19% in *nad4*L in both mtDNAs, whereas it was present at <10% in *cox1* and *cox2*. Furthermore, it was noteworthy that the cA and gA PCGs were both biased toward codons with many T residues (e.g., UUU (phenylalanine) was 13.4% in *cytb*) over those with many C residues (e.g., UCC (serine) was absent in *cytb*; **Figure 2**). This phenomenon could be associated with synonymous codon usage bias. Generally, codon bias is proposed to be the highest in regions of functional significance and is considered important for maximizing the translation efficiency [54], [55].

Interestingly, a similar trend of base bias (toward AT) was simultaneously detected in the employment of initiation and termination codons. The most frequently used initiation codon for the two *Ascaris* isolates was TTG (6 of 12 PCGs; *cox2*, *nad1–4*, and *nad6*) followed by ATT (five PCGs; *atp6*, *cox1*, *cytb*, *nad5*, and *nad4*L); furthermore, GTT (*cox3*) was used as the initiation codon (**Table S2**). Of the 12 PCGs, 10 were inferred to employ TAG (*atp6*, *cox1–3*, *nad1*, *nad3*, *nad6*, and *nad4*L) or TAA (*cytb* and *nad4*) as the termination codons, while the remaining PCGs (*nad2* and *nad5*) were deduced to end with an incomplete codon T, in accordance with studies of the other congeneric nematodes [22], [28], [30].

### Transfer RNA and ribosomal RNA genes

Twenty-two tRNAs identified in the cA and gA mtDNAs ranged from 51 to 62 bp in size and shared anticodon sequences with other previously characterized nematodes [22], [28], [30]. Their putative secondary structures exhibited strong similarities to those of other chromadorean nematodes studied thus far (see **Figure S2)** [22]–[30], [32], [39], [48]. The rRNA genes *rrn*L and *rrn*S were 960 bp and 700 bp in size for cA, respectively, and 961 bp and 700 bp in size for gA, respectively. The corresponding secondary structures were constructed and are depicted in **Figures S3 and S4** (*rrn*L and *rrn*S, respectively). The consensus secondary structures from both the genomes had an overall structural similarity with those of other nematode species in order Ascaridida. For instance, the predicted structure of *rrn*S contained 25 helical structure elements identified in *A. suum*, *B. procyonis*, and *T. canis* [27], [32], [56]. Most importantly, nucleotide locations in the *rrn*S sequences of these three ascaridoids, related to binding either the amino-acyl site (A) or the peptidyl-transferase site (P), were also recognized in the consensus secondary structure for cA and gA (**Figure S4**). Additionally, a consensus secondary structure was inferred from the 5′-region corresponding to approximately 36% of the *rrn*L in cA and gA (gray box in **Figure S3**), which has not been reported for *A. suum* or *C. elegans* [56]. This structure was similar to corresponding structures in other Ascaridida and Strongylida species [27], [32], [39], [48]. Nucleotide composition analysis revealed that the AT content of the cA and gA rRNA sequences was comparable to that in *A. lumbricoides*, *A. suum*, and *A. simplex*, but were 2.3–4.8% and 2.8–5.8% greater than that in *Baylisascaris* and *Toxocara* species, respectively (**Table S3**). Interestingly, structural comparison analysis revealed that the AT content of the cA and gA mt rRNA genes did not influence their secondary structures.

### AT-rich and non-coding regions

**Figure 3:**
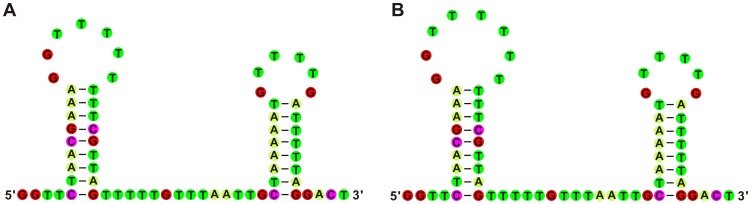
Potential hairpin structures of NCRs in mt genomes of chimpanzee *Ascaris* (A) and gibbon *Ascaris* (B). Potential hairpin structures were found within the non-coding regions (NCRs) located between the *nad4* and *cox1* genes in the mt genomes of *Ascaris* obtained from chimpanzees and gibbons. Lines indicate the inferred Watson-Crick bonds.

Two AT-rich regions of 882 bp and 886 bp were present in the mtDNAs of both cA and gA, respectively, and both possessed complex stem-loop structures (**data not shown**), as described previously for AT-rich regions of other nematodes [27], [32], [39]. Within this region, 17 (288 nt in cA) and 14 (282 nt in gA) TA dinucleotide repeat units were present. Similar repeats have been detected in the AT-rich region of other Ascaridida and Strongylida species [22]–[30], [32], [39], [48]. The function or role of these TA repeats currently remains unclear [22]. In addition, for both cA and gA, the NCR region (116 and 117 bp in length, respectively) was located between the *nad4* and *cox1* genes (**Figure 1** **and Table S2**). This region contained a 67-bp (cA) or 68-bp (gA) inverted sequence block inferred to fold into two secondary hairpin structures with 8- or 9-bp-long stems and loops that varied in length from 6–8 nucleotides (**Figure 3**). The potential stem-loop hairpin structure with a T-rich loop is hypothetically involved in the origin of second (L) strand synthesis in cestodes [57], gastropods [58], insects [59], and mammals [60].

### Comparison with other Ascaridida genomes

**Figure 4:**
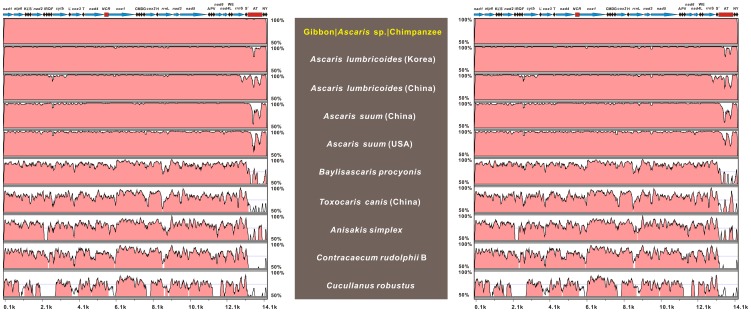
Percent identity sketch for comparisons of the 11 Ascaridida mt genomes with mVISTA. mtDNAs of *Ascaris* isolates from chimpanzee (left) and gibbon (right) were separately used as reference sequences for genome-wide comparisons carried out using the mVISTA program. The upper line denotes gene order (transcriptional direction indicated by arrows). Horizontal pink bars represent sequence similarity of the aligned regions, indicating percentage identity between 50–100% for a 100-bp moving window (shown on the Y-axis). The X-axis represents the coordinate in the mt genome. Cyan arrows (PCGs and rRNAs), black bullets (tRNAs), and red rectangles (NCR, non-coding region; AT, AT-rich region) on the upper line represent the genes or regions indicated in parentheses.

Several complete Ascaridida mt genomes are available and provide an opportunity to compare the inter- and intra-family sequence variation at the genome level. Pairwise comparisons of the PCG and rRNA genes were carried out for cA and gA mtDNAs with those of fifteen published Ascaridida species (**Table 2**). The deduced lengths of the 12 PCGs in both genomes were consistent with those of *A. lumbricoides* (China and Korea isolates), *A. suum* (China and USA isolates), and along with two rRNA genes, they were in the size range of the other eleven nematode mtDNAs (**data not shown**). The magnitude of sequence variation in each gene within both cA and gA ranged from 0.0–37.4% for nucleotide sequences and 0.0–52.7% for amino acid sequences either between cA and gA or between cA or gA and other Ascaridida species studied here. Based on the sequence divergences, among the 12 PCGs, *nad6* and *cytb* were the most variable genes whereas *cox1* and *cox2* were the most conserved. For all 12 PCGs and 2 rRNAs, the sequence divergences were found to be the lowest between cA and gA (all values ≤ 1%), followed by the divergences between cA or gA and individual species of the same family (Ascarididae), including *A. lumbricoides* (China and Korea isolates), *A. suum* (China and USA isolates), and four *Baylisascaris* species, and then the divergences between cA or gA and ascaridoid species of the Toxocaridae, Anisakidae and Cucullanidae families (see **Table 2**). Furthermore, the nucleotide sequences of the AT-rich regions in the cA and gA mtDNAs also seemed to display more divergences (all values >50%) from those of the fifteen species, excluding *A. lumbricoides* and *A. suum* (**data not shown**). Combined, these finding suggested that cA and gA may represent a same species and both were genetically more closely related to members of Ascarididae family than to members of the Toxocaridae, Anisakidae, and Cucullanidae families. This conclusion was supported by subsequent genome-wide nucleotide sequence identity analysis. As shown in **Figure 4**, the mtDNA sequence identities of the nine representatives of the order Ascaridida were plotted using the mtVISTA program with the annotation of cA or gA as references, and the respective genome-wide identities (in decreasing order) were *A. lumbricoides* (Korea isolate; 99.43% and 99.45%), *A. lumbricoides* (China isolate; 98.61% and 98.65%), *A. suum* (China isolate; 98.18% and 98.09%), *A. suum* (USA isolate; 97.45% and 97.41%), *B. procyonis* (83.48% and 83.40%), *T. canis* (China isolate; 79.13% and 79.17%), *A. simplex* (77.19% and 77.14%), *C. rudolphii* B (76.76% and 76.88%), and *C. robustus* (68.49% and 68.37%) when compared to the cA or gA, and it was noteworthy that the cA and gA genomes exhibited the highest percentage identity of 99.57%.

**Table 2:** Divergences (%) in nucleotides (left) and amino acids (right) of the protein-coding genes and rRNAs between (chimpanzee/gibbon) *Ascaris* and other Ascaridida nematodes.

**Gene**	**Chimpanzee-** ***Ascaris*** ** sp.**															
	**gA**	**ALc**	**ALk**	**ASc**	**ASu**	**Ba**	**Bp**	**Bs**	**Bt**	**TCa**	**TCc**	**Tc**	**Tm**	**Asi**	**Cru**	**Cro**
***atp6***	**0.2||0.5**	**1.0||2.5**	**0.2||0.5**	**2.0||1.5**	**1.7||0.5**	**12.7||11.6**	**12.8||8.5**	**12.0||12.1**	**13.2||12.6**	**18.8||18.1**	**19.0||18.1**	**18.2||19.1**	**17.5||17.6**	**19.3||14.1**	**18.2||15.6**	**31.7||42.0**
***cox1***	**0.3||0.2**	**0.4||0.4**	**0.4||0.4**	**0.7||0.2**	**1.8||0.2**	**9.6||9.7**	**7.6||2.3**	**9.3||2.9**	**9.0||2.9**	**12.4||5.0**	**12.4||6.2**	**12.0||4.8**	**12.1||4.9**	**14.5||7.4**	**13.9||6.7**	**20.5||15.4**
***cox2***	**0.7||0.9**	**1.0||0.9**	**0.6||0.4**	**0.1||0.4**	**2.7||1.3**	**10.4||3.9**	**9.3||3.9**	**9.9||4.7**	**10.4||3.9**	**16.0||8.9**	**15.7||8.9**	**16.2||8.5**	**15.3||9.3**	**15.7||9.9**	**16.6||8.6**	**23.3||22.0**
***cox3***	**0.4||0.4**	**0.1||0.4**	**0.3||0.8**	**1.3||0.8**	**1.8||0.8**	**11.1||5.5**	**10.8||5.9**	**10.4||5.9**	**11.6||5.9**	**16.9||8.6**	**16.8||8.6**	**16.7||8.2**	**16.7||9.8**	**18.8||11.8**	**16.5||10.6**	**29.3||32.2**
***cytb***	**0.1||0.0**	**1.9||2.7**	**0.3||0.0**	**2.0||0.8**	**2.2||1.1**	**14.0||13.3**	**13.2||11.8**	**13.6||12.8**	**13.7||12.8**	**20.4||19.8**	**20.8||22.6**	**21.5||20.7**	**22.1||20.9**	**23.2||24.9**	**24.8||25.3**	**27.7||30.1**
***nad1***	**0.2||0.3**	**0.8||1.7**	**0.2||0.7**	**2.0||0.3**	**2.1||0.3**	**13.3||10.3**	**9.6||6.9**	**12.3||10.0**	**13.3||10.3**	**17.7||16.1**	**16.3||15.2**	**16.6||13.5**	**16.8||14.1**	**17.0||13.1**	**17.2||14.5**	**24.7||30.3**
***nad2***	**0.2||0.0**	**0.7||1.1**	**0.1||0.0**	**2.1||2.1**	**2.4||2.1**	**13.0||11.7**	**11.5||11.4**	**12.0||11.0**	**12.3||10.7**	**20.1||23.5**	**19.9||21.0**	**19.1||21.7**	**19.4||20.6**	**20.5||21.5**	**25.9||28.8**	**37.4||52.7**
***nad3***	**0.0||0.0**	**0.0||0.0**	**0.0||0.0**	**0.0||0.0**	**1.8||0.9**	**10.1||7.2**	**12.2||8.1**	**10.1||7.2**	**11.6||8.1**	**15.2||17.1**	**14.9||15.3**	**14.0||10.8**	**17.3||14.4**	**16.7||15.3**	**19.1||15.3**	**29.5||33.3**
***nad4***	**0.1||0.0**	**0.6||1.0**	**0.3||0.2**	**0.3||0.5**	**2.1||0.2**	**13.0||9.3**	**13.9||8.3**	**13.4||9.3**	**13.0||9.8**	**21.7||17.4**	**21.5||17.9**	**21.2||18.3**	**22.0||17.6**	**21.7||19.1**	**25.0||22.0**	**31.5||36.7**
***nad4*** **L**	**0.9||0.0**	**1.3||1.3**	**0.4||0.0**	**0.0||0.0**	**1.3||1.3**	**11.1||2.6**	**12.4||5.2**	**9.8||3.9**	**9.4||2.6**	**21.4||11.7**	**19.7||10.4**	**18.0||13.0**	**18.4||14.3**	**19.7||14.3**	**16.7||11.7**	**27.8||31.2**
***nad5***	**0.2||0.2**	**0.1||0.0**	**0.2||0.2**	**0.1||0.0**	**1.8||0.8**	**14.3||11.7**	**13.9||10.2**	**15.3||12.1**	**13.9||11.2**	**20.9||17.6**	**20.5||17.6**	**20.1||16.5**	**19.9||16.1**	**19.8||18.2**	**21.6||20.8**	**29.7||36.9**
***nad6***	**0.5||0.7**	**0.5||0.0**	**0.5||0.0**	**0.0||0.0**	**3.0||1.4**	**15.4||13.2**	**13.3||9.7**	**14.7||12.5**	**15.4||13.2**	**23.0||24.3**	**23.2||24.3**	**20.9||20.8**	**20.0||22.2**	**21.4||19.4**	**25.5||25.7**	**30.6||42.4**
***rrn*** **S**	**0.1||-**	**3.5||-**	**0.1||-**	**0.3||-**	**0.7||-**	**10.4||-**	**8.8||-**	**10.7||-**	**10.0||-**	**19.2||-**	**18.8||-**	**18.5||-**	**20.0||-**	**16.5||-**	**21.6||-**	**30.1||-**
***rrn*** **L**	**1.0||-**	**0.9||-**	**0.6||-**	**0.5||-**	**1.1||-**	**15.1||-**	**13.1||-**	**15.2||-**	**15.2||-**	**21.6||-**	**18.6||-**	**19.6||-**	**18.6||-**	**22.0||-**	**23.0||-**	**30.9||-**
**Gene**	**Gibbon-** ***Ascaris*** ** sp.**															
	**cA**	**ALc**	**ALk**	**ASc**	**ASu**	**Ba**	**Bp**	**Bs**	**Bt**	**TCa**	**TCc**	**Tc**	**Tm**	**Asi**	**Cru**	**Cro**
***atp6***	**0.2||0.5**	**1.2||3.0**	**0.3||1.0**	**2.2||2.0**	**1.8||1.0**	**12.5||11.1**	**12.7||8.0**	**11.8||11.6**	**13.0||12.1**	**18.7||17.6**	**18.8||17.6**	**18.0||18.6**	**17.3||17.1**	**19.2||13.6**	**18.0||15.1**	**31.6||42.5**
***cox1***	**0.3||0.2**	**0.3||0.6**	**0.4||0.6**	**0.9||0.4**	**1.7||0.4**	**9.5||9.9**	**7.7||2.5**	**9.3||3.1**	**8.9||3.1**	**12.2||5.0**	**12.3||5.0**	**11.9||5.0**	**11.8||5.3**	**14.1||7.4**	**13.8||6.7**	**20.3||15.4**
***cox2***	**0.7||0.9**	**0.6||0.9**	**0.4||1.3**	**0.9||1.3**	**2.9||1.7**	**10.3||3.9**	**9.0||3.9**	**9.7||4.7**	**10.3||3.9**	**15.7||8.4**	**15.4||8.4**	**15.6||8.1**	**15.1||8.9**	**15.7||9.9**	**16.6||8.2**	**23.2||22.0**
***cox3***	**0.4||0.4**	**0.3||0.4**	**0.4||0.4**	**1.4||0.4**	**2.0||0.4**	**11.1||5.1**	**10.9||5.5**	**10.4||5.5**	**11.6||5.5**	**16.9||8.2**	**16.8||8.2**	**16.8||7.8**	**16.9||9.4**	**19.0||11.4**	**16.3||10.2**	**29.7||31.8**
***cytb***	**0.1||0.0**	**1.8||2.7**	**0.4||0.0**	**1.9||0.8**	**2.1||1.1**	**13.9||13.3**	**13.1||9.5**	**13.6||12.8**	**13.6||12.8**	**20.4||19.9**	**20.8||22.1**	**21.5||20.1**	**22.1||20.9**	**23.1||24.9**	**24.9||25.3**	**27.6||30.1**
***nad1***	**0.2||0.3**	**0.6||1.4**	**0.2||0.3**	**2.0||0.0**	**2.1||0.0**	**13.5||10.7**	**9.9||7.2**	**12.5||10.3**	**13.5||10.7**	**17.9||16.4**	**16.5||15.5**	**16.6||13.8**	**16.8||14.5**	**17.2||13.5**	**17.4||14.8**	**24.9||30.7**
***nad2***	**0.2||0.0**	**0.7||1.1**	**0.1||0.0**	**2.4||2.1**	**2.6||2.1**	**13.3||11.7**	**11.7||11.4**	**12.2||11.0**	**12.5||10.7**	**20.4||23.5**	**20.1||21.0**	**19.3||21.7**	**19.7||20.6**	**20.7||21.5**	**26.1||28.8**	**37.4||52.7**
***nad3***	**0.0||0.0**	**0.0||0.0**	**0.0||0.0**	**0.0||0.0**	**1.8||0.9**	**10.1||7.2**	**12.2||8.1**	**10.1||7.2**	**11.6||8.1**	**15.2||17.1**	**14.9||15.3**	**14.0||10.8**	**17.3||14.4**	**16.7||15.3**	**19.1||15.3**	**29.5||33.3**
***nad4***	**0.1||0.0**	**0.5||1.0**	**0.2||0.2**	**0.2||0.5**	**2.0||0.2**	**13.1||9.3**	**13.9||8.3**	**13.5||9.3**	**13.1||9.8**	**21.7||17.4**	**21.5||17.9**	**21.2||18.3**	**22.0||17.6**	**21.8||19.1**	**25.0||22.0**	**31.5||36.7**
***nad4*** **L**	**0.9||0.0**	**1.3||1.3**	**0.4||0.0**	**0.9||0.0**	**1.3||1.3**	**11.1||2.6**	**12.4||5.2**	**10.7||3.9**	**10.3||2.6**	**20.9||11.7**	**19.2||10.4**	**18.4||13.0**	**18.0||14.3**	**20.1||14.3**	**15.8||11.7**	**27.4||31.2**
***nad5***	**0.2||0.2**	**0.3||0.2**	**0.4||0.4**	**0.3||0.2**	**2.0||1.0**	**14.2||11.9**	**13.9||10.4**	**15.4||12.3**	**13.8||11.4**	**21.0||17.8**	**20.6||17.8**	**20.1||16.7**	**19.8||16.3**	**19.9||18.4**	**21.6||21.0**	**29.6||36.9**
***nad6***	**0.5||0.7**	**0.5||0.7**	**0.5||0.7**	**0.5||0.7**	**3.5||2.1**	**15.6||13.9**	**13.6||10.4**	**15.2||13.2**	**15.6||13.9**	**23.2||25.0**	**23.5||25.0**	**21.2||21.5**	**20.2||22.9**	**21.6||20.1**	**25.8||26.4**	**30.6||42.4**
***rrn*** **S**	**0.1||-**	**3.4||-**	**0.0||-**	**0.1||-**	**0.7||-**	**10.3||-**	**8.7||-**	**10.6||-**	**9.9||-**	**19.1||-**	**18.6||-**	**18.4||-**	**19.8||-**	**16.3||-**	**21.5||-**	**30.1||-**
***rrn*** **L**	**1.0||-**	**1.1||-**	**0.8||-**	**0.9||-**	**1.4||-**	**15.1||-**	**13.1||-**	**15.1||-**	**15.0||-**	**21.4||-**	**19.1||-**	**19.6||-**	**20.1||-**	**22.2||**	**22.5||-**	**30.7||-**

cA: Chimpanzee *Ascaris*, gA: Gibbon *Ascaris*, ALc: *Ascaris lumbricoides* (China isolate), ALk: *Ascaris lumbricoides* (Korea isolate), ASc: *Ascaris suum* (China isolate), ASu: *Ascaris suum* (USA isolate), Ba: *Baylisascaris ailuri*, Bp: *Baylisascaris procyonis*, Bs: *Baylisascaris schroederi*, Bt: *Baylisascaris transfuga*, TCa: *Toxocara canis* (Australia isolate), TCc: *Toxocara canis* (China isolate), Tc: *Toxocara cati*, Tm: *Toxocara malaysiensis*, Asi: *Anisakis simplex*, Cru: *Contracaecum rudolphii* B, Cro: *Cucullanus robustus*.

**Figure 5:**
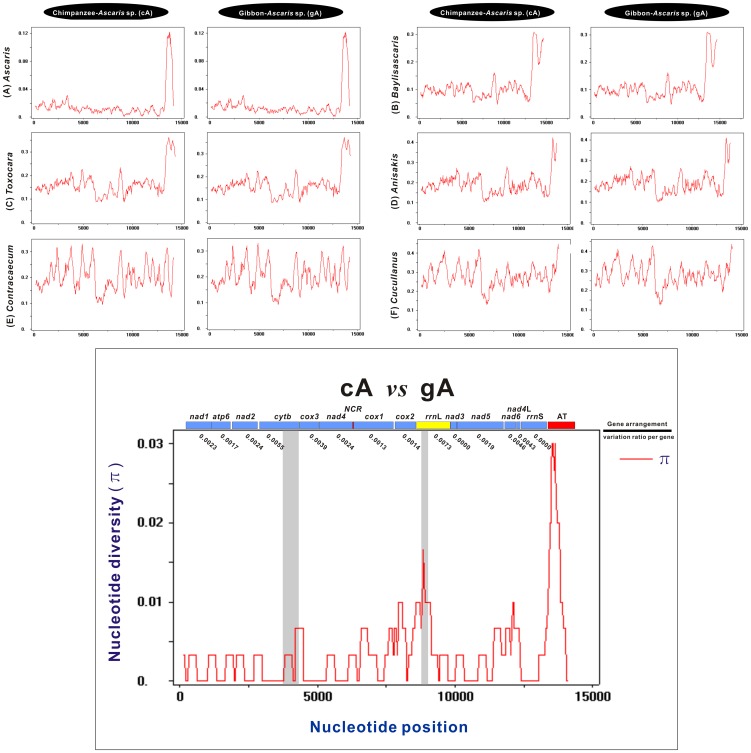
Sliding window analysis of alignments of complete mtDNAs of sequenced ascaridoid species from different genera. Sliding window analysis was separately employed to determine the nucleotide alignments of the complete mt genomes between the chimpanzee *Ascaris* (cA) or gibbon *Ascaris* (gA) and other genera. Nematodes of six genera (i.e., *Ascaris*, *Baylisascaris*, *Toxocara*, *Anisakis*, *Contracaecum*, and *Cucullanus*) were analyzed. The red line indicates the value of nucleotide diversity Pi (π) in a sliding window of 300 bp with a step size of 30, and the value is inserted at its mid-point. Each gene boundary coupled with a variation ratio per gene is indicated only in the plot of the nucleotide diversity between cA and gA. Gene regions used previously by Xie et al. (2011) are also denoted by shaded columns in this plot.

In general, pairwise analysis-based sliding window between species can aid to mirror the overall pattern of diversity across all tested species [61]. To clearly explore the diversities within and between mt genes and serve the following identification of molecular markers, sliding window analysis was also employed in the complete nucleotide alignments of cA and gA mtDNAs and 15 other Ascaridida species (**Figure 5**). The nucleotide diversity π for each gene (including the NCRs) in alignments either between cA or gA and each of the six genera appeared to gradually increase regardless of the range ability (**Figures 5A** to **F**). The greatest π value within every curve was detected in the AT-rich region, which may be because this region was the most variable part of the genome both in terms of length and nucleotide sequence [3], [10]. In contrast with the AT-rich region, the π values in the 12 PCGs and 2 rRNAs were complex and regular. Specifically, (i) the π values in the 12 PCGs and 2 rRNAs between cA and gA in six genera were essentially similar; (ii) the π values of PCGs or rRNAs with high sequence variability significantly increased in the following order: *Ascaris* < *Baylisascaris* < *Toxocara* < *Anisakis* < *Contracaecum* < *Cucullanus*; (iii) the genes with pronounced peaks and troughs seemed to possess higher sequence variability than the others (e.g., *cytb* and *nad6*); and (iv) the most or least variable genes remained constant across all curves. To a certain extent, the observations further reinforced the conclusion that cA and gA represent a single species that shared the closest relationship with the *Ascaris* species within Ascaridida.

In addition, in the sliding window analysis of the nucleotide alignment of both cA and gA mtDNAs, the inter- and intra-gene nucleotide diversity was relatively low, and π ranged from 0 to 0.167 (**Figure 5**). However, when matched with the calculation of the number of variable positions per unit length of gene, *rrn*L, *cytb*, *nad6*, and *nad4*L showed high sequence divergence, and *rrn*S, *nad3*, *cox1*, and *cox2* showed low or no sequence divergence. This finding was dramatically in agreement with the results from pairwise comparisons made among the nucleotide and/or amino acid sequences from the PCGs and rRNAs in cA and gA mtDNAs and the sequence data of the fifteen published nematodes, further suggesting that many alternative genes remain to be identified and considered as new molecular markers for molecular ecology, biogeographical analysis, population genetics, and diagnostics. For example, genes with high sequence variability could offer an opportunity to provide greater resolution in species-specific identification and differentiation of some zoonotic Ascaridida nematodes. Dangoudoubiyam et al. (2009) and Gatcombe et al. (2010) relied on the limited multiple sequence alignment of *cox2* available at the time, and developed species-specific oligonucleotide primers to target regions of variability best suited to differentiate *B. procyonis* from *T. canis*, *T. cati*, *A. suum*, and *Toxascaris leonine* [62], [63]. Although this gene, particularly when combined, provided adequate resolution for their studies, the sliding window analysis conducted here indicated that *cox2* was amongst the most conserved gene in Ascaridida, rendering primer design and amplification of this gene relatively easy, but missing the very signal pursued. It is noteworthy that many more mtDNA genes (e.g., *rrn*L, *cytb* and *nad6*; *rrn*L and *cytb* were also described in ref. [27]) were uncovered in this study as potential candidates for use in studies of phylogenetics and population genetics, in addition to species-specific identification and diagnostics, due to their higher variability and more informative character. Perhaps these markers can be further validated when additional secernentean mtDNAs become available, especially from the order Ascaridida.

### Identification of molecular markers

Genes/regions possessing good sequence divergence were identified during mitochondrial genome-wide comparative analysis and sliding window analysis, and they could be suitable as new mt makers (fragments) for species-specific identification. To test the genes/regions that could be applied to species characterization and differentiation in Ascaridida, all of the genes/regions of the 17 genomes that could be aligned and exhibited marked sequence variability (**Figures 4** and **5**), as well as the regions previously reported for the specific identification of *B. procyonis* [27], were extracted. These fragments were then used for species-specific identification using conventional PCR. Three targeted regions (namely, Asc-cI, Asc-nII, and Asc-rIII) from the *cytb*, *nad6*, and *rrn*L genes, respectively, were found to contain abundant variable sites (Vn/size values of 0.46, 0.71, and 0.46, respectively) and could be used for the following PCR assays. The corresponding PCR primer pairs were subsequently designed to target a 480-bp fragment in Asc-cI, a 150-bp fragment in Asc-nII, and a 638-bp fragment in Asc-rIII after manual inspection of these targeted region sequences. The primer pairs were as follows: for Asc-cI, forward: 5′-TTAggAgTgATTAAgTTgg-3′ and reverse: 5′-TTAgATTCTAgTTATAATAgCAAAA-3′; for Asc-nII, forward: 5′-TACTAAAACTTAgAATggTAATgTAgTg-3′ and reverse: 5′-TCCATCATCATTgAAAATACTCTAA-3′; and for Asc-rIII, forward: 5′-TTTAATTggATggTTATg-3′ and reverse: 5′-CAATTACgACTATgACCTTAC-3′. The specificities of these three primer pairs were tested by PCR of the mitochondrial DNA from cA, gA, *A. lumbricoides*, *A. suum*, *T. canis*, *T. leonine*, and four *Baylisascaris* spp. (namely, *B. schroederi*, *B. ailuri*, *B. transfuga*, and *B. procyonis*). Interestingly, the three targeted amplification bands (480-bp Asc-cI, 150-bp Asc-nII, and 638-bp Asc-rIII) were consistently present in cA, gA, *A. lumbricoides*, and *A. suum* but not in the other ascaridoids tested (**data not shown**). The PCR results clearly indicated that these three fragments could be readily used to differentiate cA and gA strains from other related ascaridoids, excluding the two congeners (*A. lumbricoides* and *A. suum*). Further nucleotide sequence analysis of the three fragment regions of cA, gA, *A. lumbricoides*, and *A. suum* revealed high sequence similarity among these strains for each region, and it was found that they differed by only 19 bases in Asc-cI, 8 bases in Asc-nII, and 10 bases in Asc-rIII (**Figure S5**). Therefore, the PCR assays developed in this study cannot differentiate between cA or gA and *A. lumbricoides* and *A. suum*. The existence of significant morphological and biological similarities across ascaridoid nematodes, particularly in *Ascaris* spp., is well known [28], and these similarities render traditional identification and diagnosis (based on morphological features alone) of the parasitic species time-consuming and difficult. Recently, Nejsum et al. (2010) used mt markers (e.g., *cox1*) in combination with PCR to successfully identify the roundworm species in zoo chimpanzees, and the roundworm was identified as *A. suum* [19]. However, based on our genome-wide comparative analysis and sliding window analysis, *cox1* was the most conserved gene amongst the Ascaridida mtDNA dataset, with lower nucleotide divergence than other genes such as *cytb*, *nad6*, and *rrn*L. Most importantly, due to high similarities still in the *cytb*, *nad6*, and *rrn*L genes of *Ascaris* species, our attempts to differentiate cA from *A. suum* and *A. lumbricoides* failed. In light of the discrepancy between the conclusion drawn by Nejsum et al. [19] and our PCR species-specific identification results, we believe that the advent of next-generation sequencing (NGS) will facilitate future nuclear genomics studies to provide novel insights into the accurate identity of cA and gA. Furthermore, our PCR assays revealed other faint bands such as a 200-bp band in cA, gA, *A. lumbricoides*, and *A. suum* and multiple bands in *B. transfuga* (**not shown**); these bands may be associated with the relative specificity of the three primer pairs.

### Substitution ratios

**Figure 6:**
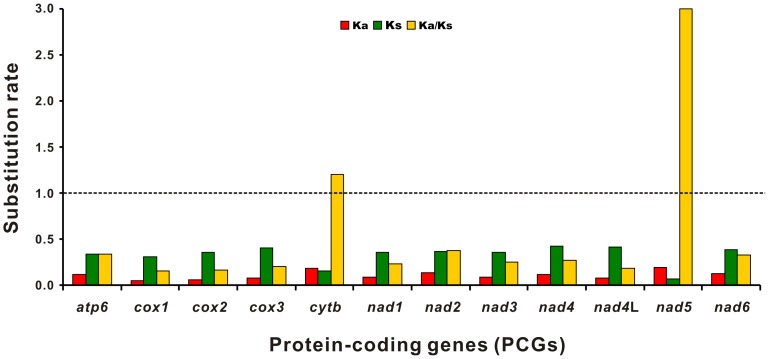
Evolutionary rates in the Ascaridida mtDNAs. The rate of nonsynonymous substitutions (Ka), the rate of synonymous substitutions (Ks), and the ratio of the former to the latter (Ka/Ks) for each protein-coding gene (PCG) are shown.

Estimation of synonymous (Ks) and nonsynonymous (Ka) substitution rates is important to understand the dynamics of molecular sequence evolution [64], and the Ka/Ks ratio can be used as an indicator of selective pressure acting on PCGs in evolution and the ratio is indicative of negative or purifying selection (Ka/Ks < 1), neutral mutation (Ka/Ks  =  1), and positive or diversifying selection (Ka/Ks > 1) [65], [66]. To analyze the selection pressure on mitochondrial PCGs of the Ascaridida species, Ka, Ks, and the Ka/Ks ratio were calculated for 12 PCGs from cA and gA and 15 other nematode isolates. The PCG *nad5* exhibited the highest ratio, followed by *cytb*, whereas *cox1* had the lowest ratio (**Figure 6**). Of the 12 PCGs, the Ka/Ks ratios of ten genes (*atp6*, *cox1–3*, *nad1–4*, *nad4*L, and *nad6*) were <1 (0.157*–*0.377), suggesting that these genes are evolving under negative or purifying selection [66]. The Ka/Ks ratios of the remaining two genes were 1.206 (*cytb*) and 2.998 (*nad5*), respectively, indicating that the two genes have evolved under positive or diversifying selection [65].

### Phylogeny

**Figure 7:**
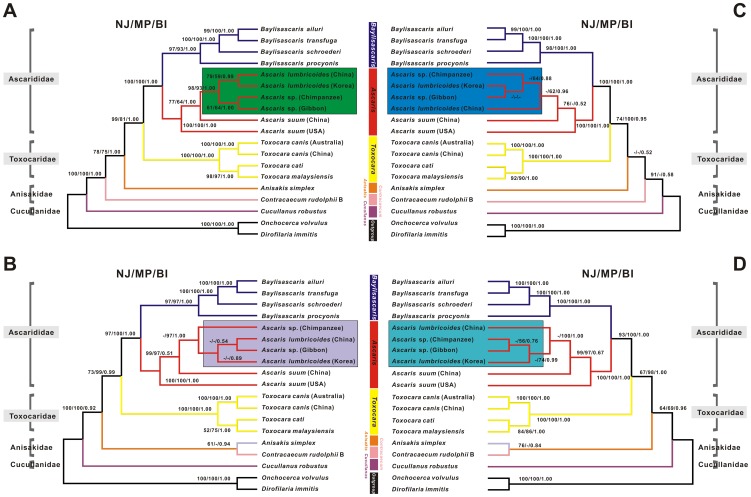
Phylogenetic relationships among ascaridoid nematodes. Phylogeny was inferred from neighbor-joining (NJ), maximum parsimony (MP), and Bayesian inference (BI) analyses, based on mt data (i.e., concatenated amino acid sequences of 12 PCGs (A), concatenated nucleotide sequences of 12 PCGs (B), pooled nucleotide sequences (C) for rRNAs and tRNAs, and pooled nucleotide sequences (D) of all PCGs and RNAs) of 17 Ascaridida species for which complete mtDNA sequences are available, using two filarial species (*D. immitis* and *O. volvulus*) as the outgroups. The numbers along the branches indicate bootstrap values resulting from different analyses in the order NJ/MP/BI.

The mtDNA sequence of Ascaridida is a useful resource for studying its taxonomic status and for analyzing the evolutionary relationship within this order [24], [26]–[28], [31]. Here, four separate mt genome datasets were used to infer phylogenic data. After concatenation or pooling, one amino acid sequence alignment (inferred from 12 PCGs) and three nucleotide sequence alignments (derived from 12 PCGs, both RNAs (2 rRNAs + 22 tRNAs), and all PCGs and RNAs) from the 17 species were separately used to reconstruct the phylogenetic relationships based on NJ, MP, and BI analyses as follows (see **Figure 7**):

**(i) Phylogenetic analysis of mitochondrial proteins.** As shown in **Figure 7A**, all phylogenetic analyses (NJ/MP/BI) of the concatenated amino acid data clearly supported the distinct classification positions of *Ascaris* and *Baylisascaris* (family Ascarididae), *Toxocara* (family Toxocaridae), *Anisakis* (family Anisakidae) and *Contracaecum* (family Cucullanidae) in the order Ascaridida, each as a monophyletic sister group with high statistical support (all bootstrap values ≥75 or  = 1.00). cA and gA as one group in the branch were paraphyletic with two *A. lumbricoides* isolates, and formed the sister taxon to two *A. suum* isolates within *Ascaris*. All nodes had moderate and/or strong bootstrap support, suggesting that cA and gA may represent a single species, which was more closely related to *A. lumbricoides* than to *A. suum*. This finding was consistent with the results of previous morphological and molecular biology studies [13]–[15], [17]. Surprisingly, unlike the two *A. lumbricoides* isolates, the *A. suum* Chinese isolate formed one independent monophyletic group with the *A. suum* USA isolate (see **Figure 7A**); and this result was similar to that observed by Liu et al. (2012) [28]. Currently the potential factors contributing to this phenomenon remain unknown. Future large-scale studies using mt protein data from *A. suum* isolates worldwide are required to ascertain these intraspecific relationships. Moreover, with respect to the inter-relationships of *B. ailuri*, *B. procyonis*, *B. schroederi*, and *B. transfuga* in *Baylisascaris*; two *T. canis* isolates, *T. cati*, and *T. malaysiensis* in *Toxocara*; and the *Anisakis* species *A. simplex* and the *Contracaecum* species *C. rudolphii* B in the family Anisakidae, their topologies were congruent with previously proposed molecular phylogeny based on the mtDNA dataset [24], [27], [29].

**(ii) Phylogenetic analysis of mitochondrial DNA.** Data from the three identical trees (NJ/MP/BI) produced by concatenated nucleotide sequences of 12 PCGs (**Figure 7B**) or pooled nucleotide sequences of tRNAs and rRNAs (**Figure 7C**) and of all PCGs and RNAs (**Figure 7D**) were similar to the results of the amino acid data, with the exception of the relative positions of cA, gA and two *A. lumbricoides* isolates in the genus *Ascaris* and the relative relationships of *A. simplex* and *C. rudolphii* B. gA was clustered with *A. lumbricoides* (China isolate), and then grouped with *A. lumbricoides* (Korea isolate) in a branch that was paraphyletic with cA (**Figure 7B**). *A. simplex* and *C. rudolphii* B were also simultaneously grouped together. However, reverse topologies were found, wherein gA was grouped with *A. lumbricoides* (Korea isolate) and then grouped with cA in a branch that was paraphyletic with *A. lumbricoides* (China isolate; **Figure 7C**). *A. simplex* and *C. rudolphii* B also seemed to form two independent monophyletic groups, similar to the results shown in **Figure 7A**. For the *Ascaris* topology in **Figure 7D**, cA and gA were grouped together with *A. lumbricoides* (Korea isolate), which then formed a branch that was paraphyletic with *A. lumbricoides* (China isolate). Interestingly, *A. simplex* and *C. rudolphii* B were clustered together again, consistent with in the results shown in **Figure 7B**. It was noteworthy that these three putative *Ascaris* topologies did not appear to be supported by the strong bootstraps from the NJ analysis in all trees (all values <50). Similarly, the relationships of *A. simplex* and *C. rudolphii* B did also not received conspicuous confidence levels from the MP analysis in these trees. Combined with the phylogenies inferred from the nucleotide data, however, the highly unstable inter-relationships among *Ascaris* seemed prone to develop a unified conclusion: both cA and gA shared the closest relationship with *A. lumbricoides*, regardless of isolate origins, which was in agreement with the inferences from (i) section.

Taken together, our phylogenetic analyses coupled with the pairwise comparisons of nucleotide and/or amino acid sequences from the PCGs rRNAs as well as the genome-wide identity analysis provided strong statistical support that the cA and gA isolates described here may not only represent the same species but they also share genetic similarity with *A. lumbricoides*. Caution is warranted when this conclusion was drawn based on the mtDNA dataset, because mtDNA sequences may be influenced by hybridization and introgression, potentially challenging the classification of organisms [28]. In addition, phylogenetic analyses revealed that *Ascaris* was more closely related to *Baylisascaris* than the other genera within the order Ascaridida (**Figure 7**), which was consistent with previously reported findings [16], [27], [67], [68]. However, relationships between any of the *Ascaris* spp., *Baylisascaris* spp., *Toxocara* spp., *Anisakis* spp., and *Contracaecum* spp. and *Cucullanus* spp. were undefined in the NJ, MP, or ML analyses (without bootstrap support) (see **Figure 7**). Therefore, in-depth and larger studies of the evolutionary relationships among taxa in Ascaridida using mtDNA data are required to ascertain the abovementioned hypothesis. Recent advances in the high-throughput techniques for mtDNA sequencing provide a platform for the in-depth phylogenetic analysis of the order Ascaridida [21].

In summary, this study was the first to sequence the complete mt genomes of two representative *Ascaris* isolates derived from non-human primates, chimpanzees and gibbons. Both mtDNAs were equipped with a typical chromadorean mitogenome structure and featured extremely high synteny with Ascaridida nematodes, particularly with *Ascaris* species. When compared with the other ascaridoid mtDNAs, each genome exhibited notable levels of AT and GC skew. Further pairwise comparisons of amino acid and nucleotide sequences, genome-wide nucleotide sequence identity analysis, and phylogeny revealed that cA and gA represented the same species and shared the closest relationship with *A. lumbricoides*, suggesting that both these isolates is a single species genetically similar to *A. lumbricoides*. This finding should enhance public alertness to roundworms in chimpanzees and gibbons. Furthermore, the mtDNA data presented here enrich the resource of markers for molecular diagnostic, systematic, population genetic, and evolutionary biological studies of parasitic nematodes of socioeconomic importance from wild or domestic hosts.

## Supporting Information

Figure S1 **Synteny between the mtDNAs of** ***Ascaris*** **from chimpanzees and gibbons and other selected ascaridoid nematodes.** Synteny blocks were designed using chimpanzee or gibbon *Ascaris* sp. as the origins. The gene order for chimpanzee *Ascaris*, gibbon *Ascaris*, *Ascaris lumbricoides*, *Anisakis simplex*, *Baylisascaris procyonis*, *Contracaecum rudolphii* B, and *Toxocara canis* is completely conserved, illustrating their close relationship.
(TIF)

Figure S2 **Inferred secondary structures for 22 tRNAs in chimpanzee** ***Ascaris*** **(A) and gibbon** ***Ascaris*** **(B) mtDNAs.**
(TIF)

Figure S3 **Inferred consensus secondary structure for the** ***rrn*** **L gene in chimpanzee and gibbon** ***Ascaris*** **mtDNAs.** Nucleotide substitutions in the gibbon *Ascaris* mt-*rrn*L gene sequence are indicated by cyan circled nucleotides, and insertions/deletions are indicated by arrows. Red indicates nucleotides showing 100% identities and blue indicates ≥75% identities. Base pairing is indicated as follows: Watson-Crick pairs by lines, wobble GU pairs by large dots, and other non-canonical pairs by small dots. Binding sites for the amino-acyl trn (A), peptidyl-transferase (P), or both (AP) [56] are indicated by lines. The gray box highlights the consensus secondary structure inferred from 5′ 342 nucleotides of the *rrn*L gene in the *Ascaris* mtDNAs of chimpanzees and gibbons, which is not reported for *A. suum* or *C. elegans*.
(TIF)

Figure S4 **Inferred consensus secondary structure for the** ***rrn*** **S genes in chimpanzee and gibbon** ***Ascaris*** **mtDNAs.** Red indicates nucleotides showing 100% identities and blue denotes ≥75% identities. Symbols for base pairings are as the same as those used in Figure S3. Conserved secondary structure elements are denoted by bold numbers (1–48) [39]. Lines indicate the binding sites for the amino-acyl trn (A) or peptidyl-transferase (P) [56].
(TIF)

Figure S5 **Sequence similarities of three mt fragment regions (Asc-cI, Asc-nII, and Asc-rIII) from six** ***Ascaris*** **species.** Three mt gene regions, namely, Asc-cI, Asc-nII, and Asc-rIII, that were identified as molecular markers for species-specific identification and diagnosis in Ascaridida were separately aligned among the chimpanzee *Ascaris*, gibbon *Ascaris*, *A. lumbricoides* (China isolate), *A. lumbricoides* (Korea isolate), *A. suum* (China isolate), and *A. suum* (USA isolate) using ClustalX with manual adjustment. Red indicates base showing 100% identities and blue indicates ≥75% identities.
(PDF)

Table S1 **List of oligonucleotide primers for PCR amplification of the 12 fragments from gibbon and chimpanzee** ***Ascaris*** **and their positions in gibbon** ***Ascaris*** **mtDNA.**
(DOC)

Table S2 **Annotation of the mitochondrial genomes of chimpanzee** ***Ascaris*** **(cA) and gibbon** ***Ascaris*** **(gA).** ^a^The inferred lengths of the amino acid sequences of 12 protein-coding genes. ^b^Negative numbers indicate the overlap of adjacent genes. Ini/Ter codons: initiation and termination codons; nt: nucleotide; aa: amino acid.
(DOC)

Table S3 **Size and nucleotide composition of different genomic regions in 11 ascaridoids reported within Ascaridida.** ^a^All protein-coding genes were accounted for. ^b^In base pairs.
(DOC)
