# Supplementary figures and images for: Complete Mitochondrial Genomes of Chimpanzee- and Gibbon-Derived Ascaris Isolated from a Zoological Garden in Southwest China
Source: PLoS One. 2013 Dec 17;8(12):e82795. doi: 10.1371/journal.pone.0082795 (PMC3866200; doi:10.1371/journal.pone.0082795)

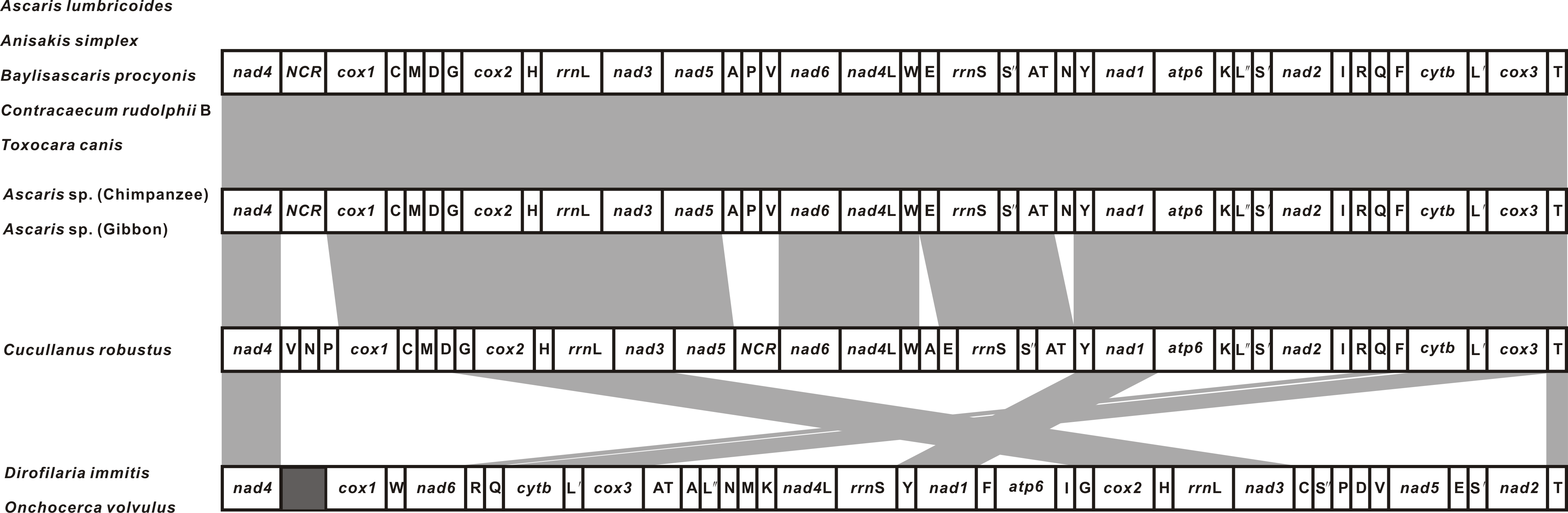

Supplement: Figure S1 — Synteny between the mtDNAs of Ascaris from chimpanzees and gibbons and other selected ascaridoid nematodes. Synteny blocks were designed using chimpanzee or gibbon Ascaris sp. as the origins. The gene order for chimpanzee Ascaris, gibbon Ascaris, Ascaris lumbricoides, Anisakis simplex, Baylisascaris procyonis, Contracaecum rudolphii B, and Toxocara canis is completely conserved, illustrating their close relationship. (TIF) [file pone.0082795.s001.tif]

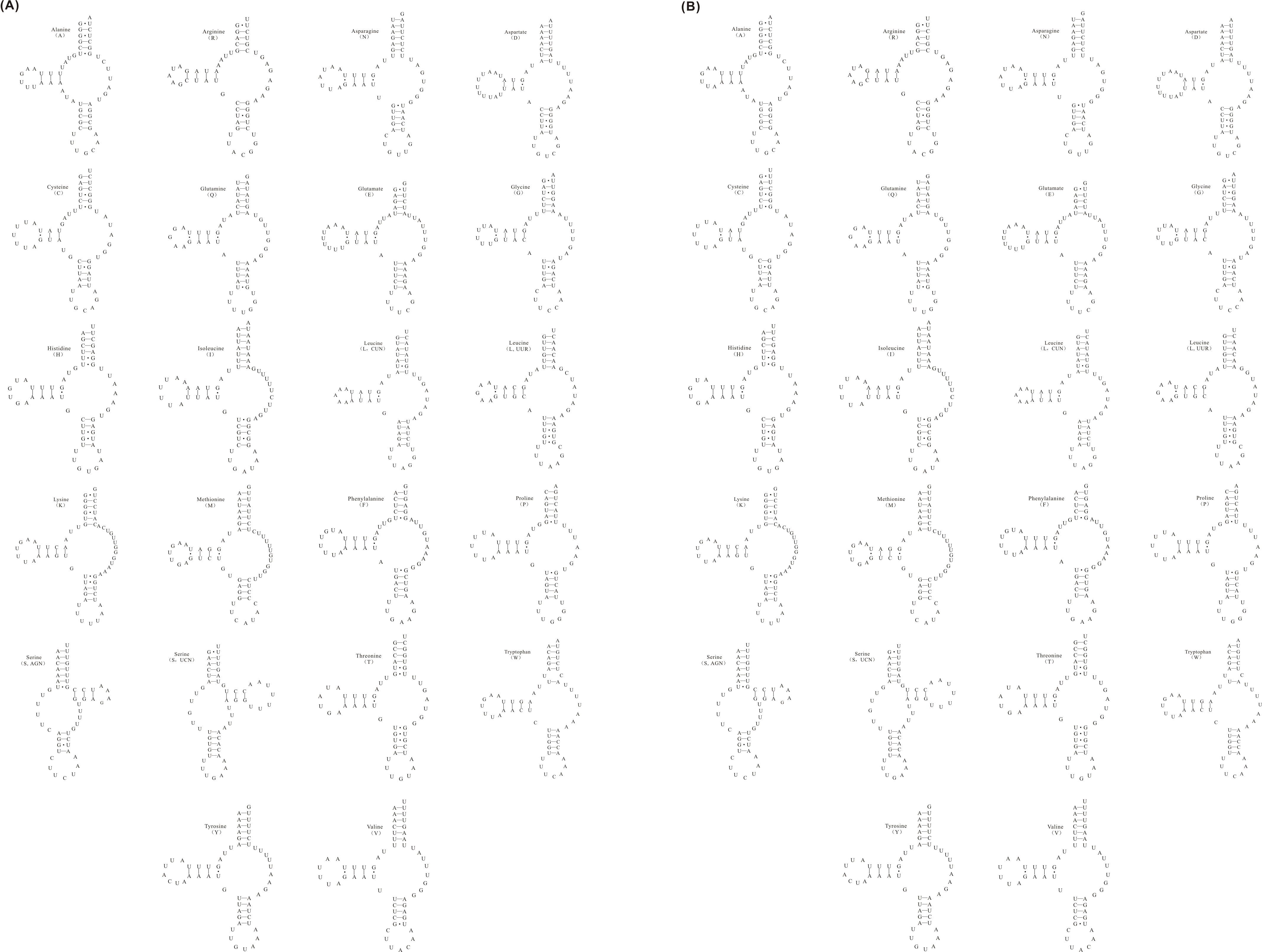

Supplement: Figure S2 — Inferred secondary structures for 22 tRNAs in chimpanzee Ascaris (A) and gibbon Ascaris (B) mtDNAs. (TIF) [file pone.0082795.s002.tif]

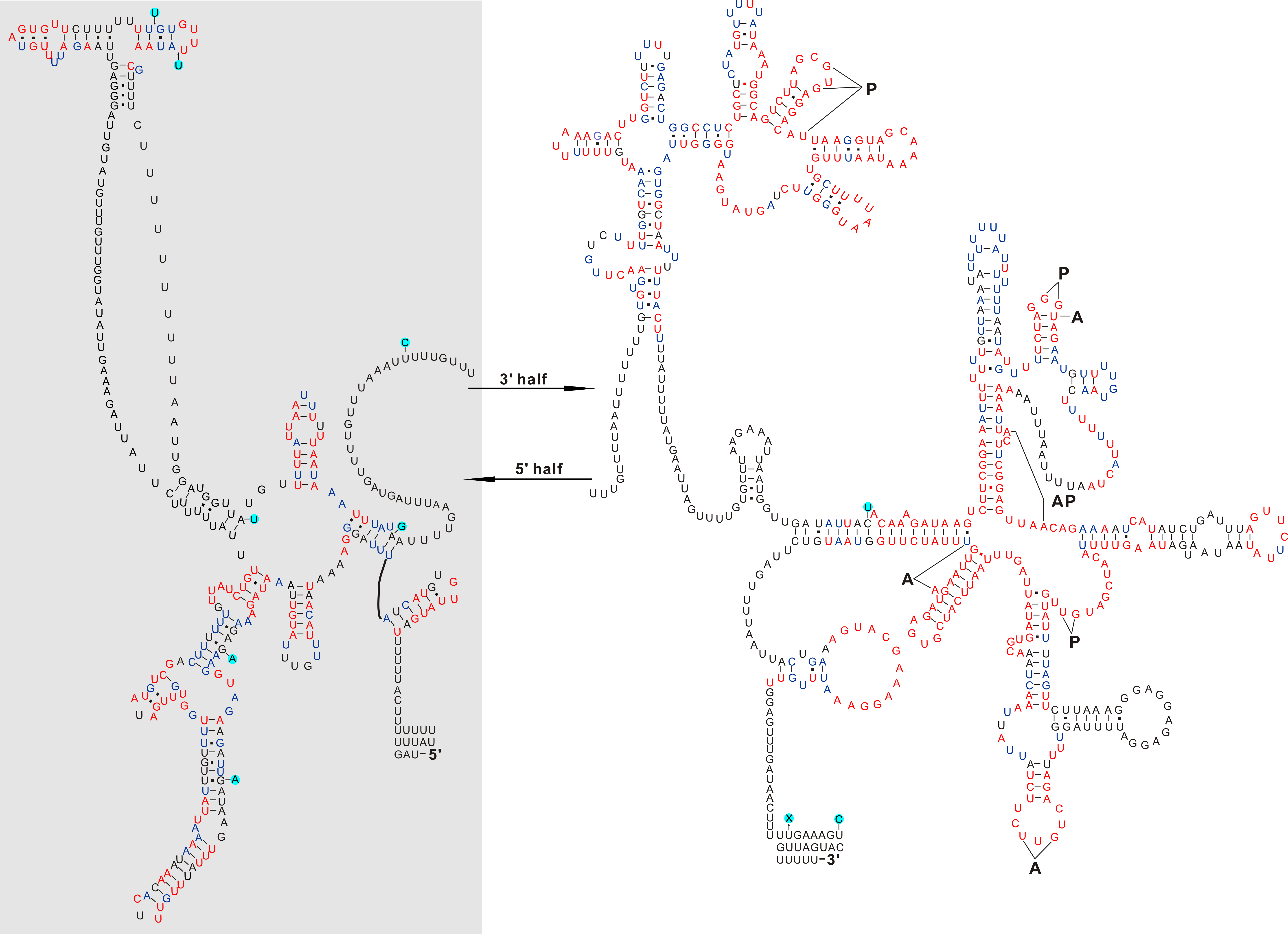

Supplement: Figure S3 — Inferred consensus secondary structure for the rrn L gene in chimpanzee and gibbon Ascaris mtDNAs. Nucleotide substitutions in the gibbon Ascaris mt-rrnL gene sequence are indicated by cyan circled nucleotides, and insertions/deletions are indicated by arrows. Red indicates nucleotides showing 100% identities and blue indicates ≥75% identities. Base pairing is indicated as follows: Watson-Crick pairs by lines, wobble GU pairs by large dots, and other non-canonical pairs by small dots. Binding sites for the amino-acyl trn (A), peptidyl-transferase (P), or both (AP) [56] are indicated by lines. The gray box highlights the consensus secondary structure inferred from 5′ 342 nucleotides of the rrnL gene in the Ascaris mtDNAs of chimpanzees and gibbons, which is not reported for A. suum or C. elegans. (TIF) [file pone.0082795.s003.tif]

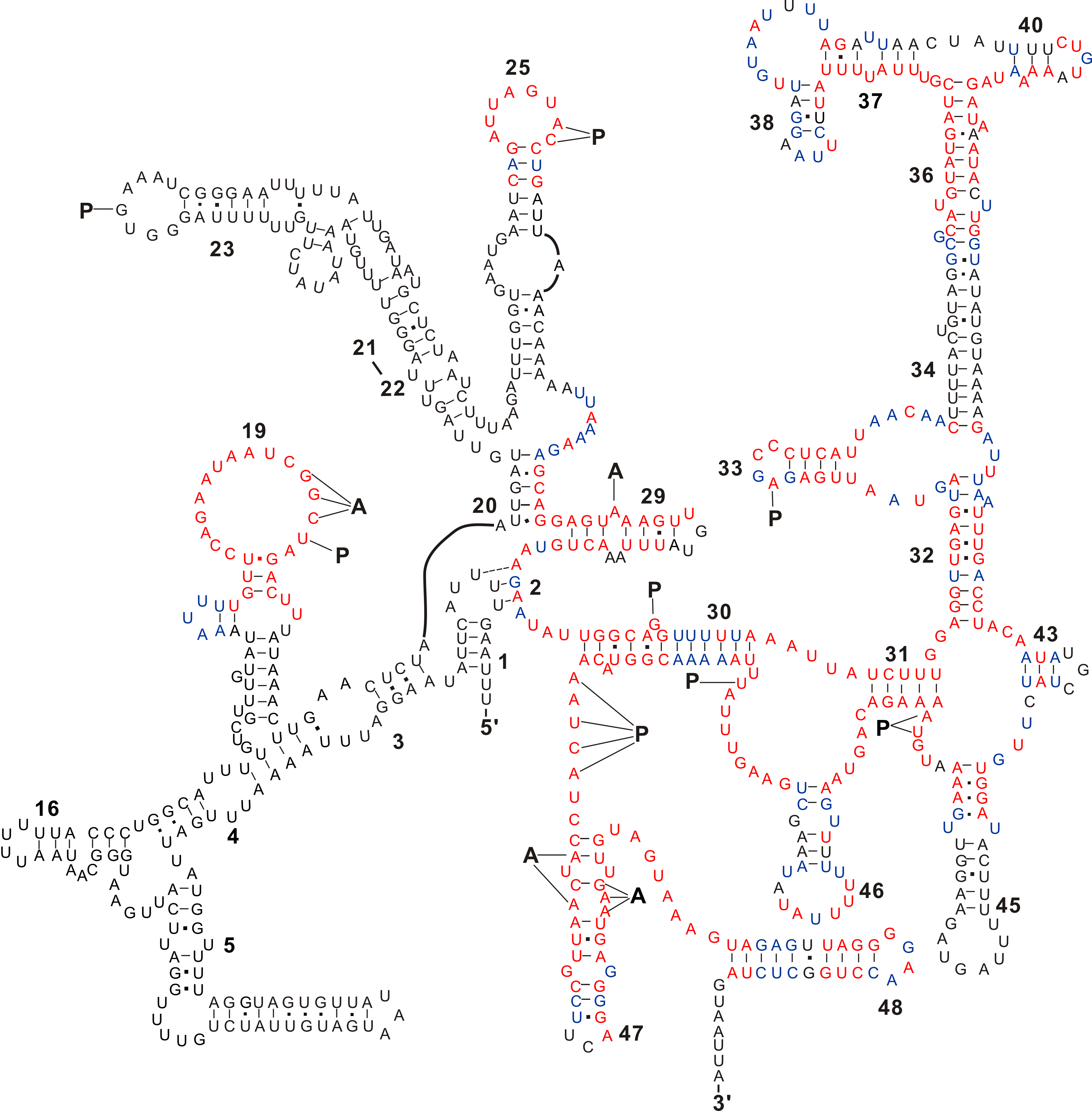

Supplement: Figure S4 — Inferred consensus secondary structure for the rrn S genes in chimpanzee and gibbon Ascaris mtDNAs. Red indicates nucleotides showing 100% identities and blue denotes ≥75% identities. Symbols for base pairings are as the same as those used in Figure S3. Conserved secondary structure elements are denoted by bold numbers (1–48) [39]. Lines indicate the binding sites for the amino-acyl trn (A) or peptidyl-transferase (P) [56]. (TIF) [file pone.0082795.s004.tif]
