# Supplementary material for: Complete Mitochondrial Genomes of Chimpanzee- and Gibbon-Derived Ascaris Isolated from a Zoological Garden in Southwest China
Source: PLoS One. 2013 Dec 17;8(12):e82795. doi: 10.1371/journal.pone.0082795 (PMC3866200; doi:10.1371/journal.pone.0082795)
Supplement: Figure S5 — Sequence similarities of three mt fragment regions (Asc-cI, Asc-nII, and Asc-rIII) from six Ascaris species. Three mt gene regions, namely, Asc-cI, Asc-nII, and Asc-rIII, that were identified as molecular markers for species-specific identification and diagnosis in Ascaridida were separately aligned among the chimpanzee Ascaris, gibbon Ascaris, A. lumbricoides (China isolate), A. lumbricoides (Korea isolate), A. suum (China isolate), and A. suum (USA isolate) using ClustalX with manual adjustment. Red indicates base showing 100% identities and blue indicates ≥75% identities. (PDF) [file pone.0082795.s005.pdf]

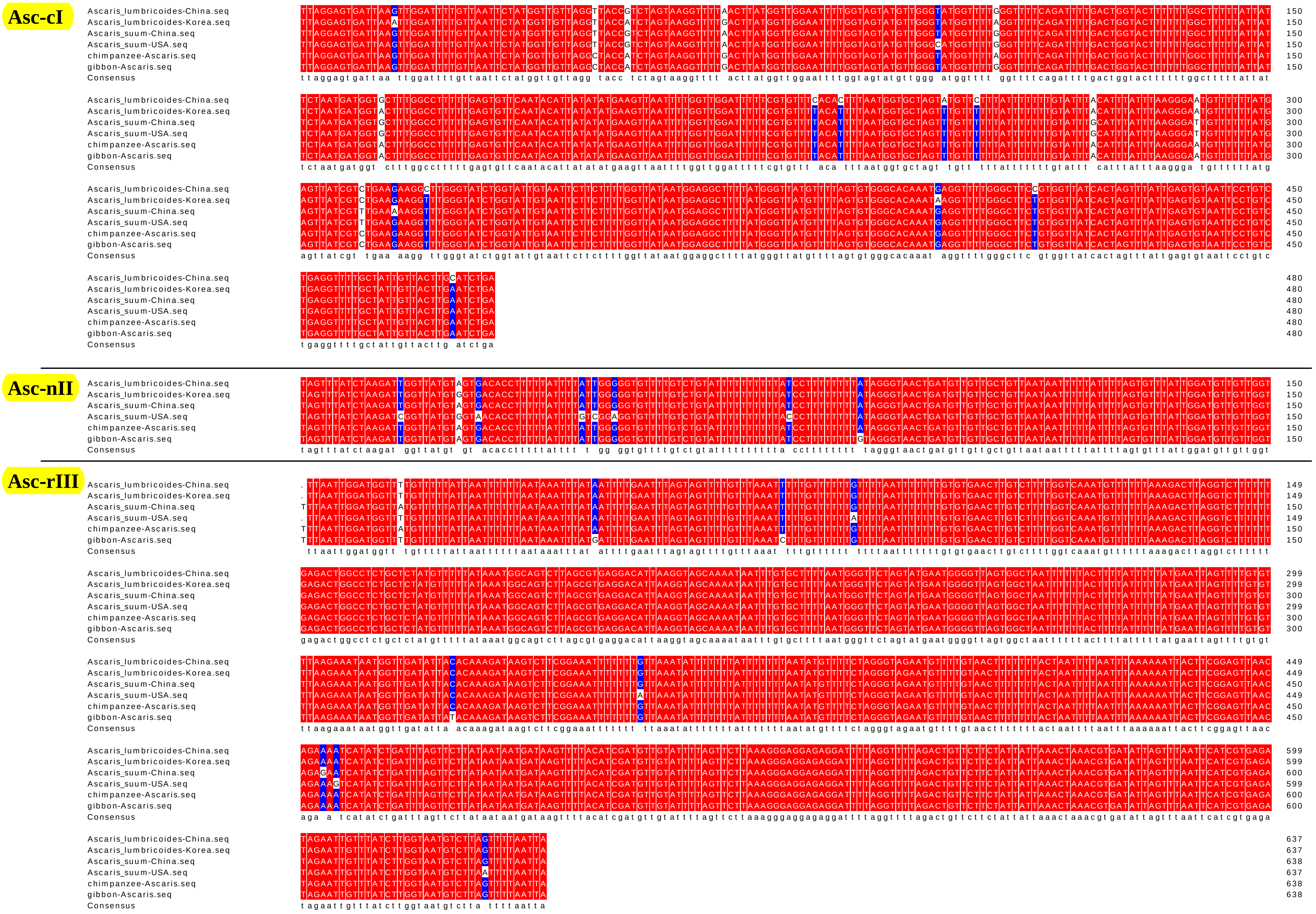
Asc-cI
Asc-nII
Asc-rIII
Ascaris_lumbricoides-China.seq
Ascaris_lumbricoides-Korea.seq
Ascaris_suum-China.seq
Ascaris_suum-USA.seq
chimpanzee-Ascaris.seq
gibbon-Ascaris.seq
Consensus
