# Supplementary material for: Complete Mitochondrial Genomes of Chimpanzee- and Gibbon-Derived Ascaris Isolated from a Zoological Garden in Southwest China
Source: PLoS One. 2013 Dec 17;8(12):e82795. doi: 10.1371/journal.pone.0082795 (PMC3866200; doi:10.1371/journal.pone.0082795)
Supplement: Table S1 — List of oligonucleotide primers for PCR amplification of the 12 fragments from gibbon and chimpanzee Ascaris and their positions in gibbon Ascaris mtDNA. (DOC) [file pone.0082795.s006.doc]

**Table S1. List of oligonucleotide primers for PCR amplification of the 12 fragments from gibbon and chimpanzee *Ascaris* and their positions in gibbon *Ascaris* mtDNA.**

| **Fragments and Primer ID** | **Location on gibbon *Ascaris* mtDNA (gene region)** |  | **Sequence (5' to 3')** |  | **Expected amplicon size** |
| --- | --- | --- | --- | --- | --- |
| **mtDNA 1 fragment AlP-1** | **14,053-14,073 (*cox3*) 1,423-1,443 (*cox1*)** |  | **Forward: ATGTGTTGTTTGGTGGATTAT Reverse: CAAACCAAACAAAAAATACAA** |  | **1, 665 bp** |
|
| **mtDNA 2 fragment AlP-2** | **1,060-1,086** **(*nad4*) 3,022-3,046 (*tRNA*-Met)** |  | **Forward: TTTTGGGTGTTATTTGTTTATTTTTTC Reverse: TCAATAAGAGAAAAACACCAAGAGG** |  | **1, 989 bp** |
|
| **mtDNA 3 fragment AlP-3** | **2,756-2,778 (*cox1*) 4,070-4,094 (*rrn*L)** |  | **Forward: TTGCTTTGTTTTTGTTTATTTAT Reverse: TAAAAAAAAATAAACAGATAACAAA** |  | **1, 342 bp** |
|
| **mtDNA 4 fragment AlP-4** | **3,847-3,868 (*cox2*) 4,983-5,004 (*nad3*)** |  | **Forward: GGGTTATTGAATGATTAGAAGC Reverse: ACCACACTCAAAAGAAGAAATC** |  | **1, 157 bp** |
|
| **mtDNA 5 fragment AlP-5** | **4,705-4,724 (*rrn*L) 5,554-5,578 (*nad5*)** |  | **Forward: GGGAGGAGAGGATTTTAGGT Reverse: TGTAAAACAAAACTAAAAAAAAACT** |  | **875 bp** |
|
| **mtDNA 6 fragment AlP-6** | **5,464-5,487 (*nad5*) 6,846-6,867 (*tRNA*-Ala–*tRNA*-Pro)** |  | **Forward: TTACTTGTTTTTGTGGGTAGAATG Reverse: CCTACTGCATAGAGCCAGAATC** |  | **1, 404 bp** |
|
| **mtDNA 7 fragment AlP-7** | **6,605-6,627 (*nad5*) 8,317-8,340 (*rrn*S)** |  | **Forward: AGTTTTTGGTTGACTTTTTTGCT Reverse: CTGTCTTTTACATTTTCAACCTTC** |  | **1, 724 bp** |
|
| **mtDNA 8 fragment AlP-8** | **8,107-8,133 (*rrn*S) 10,286-10,311 (*nad1*)** |  | **Forward: GCAGGTTTTTAAATTATCTTTGGAGGT Reverse: GCCCTACGAATAAACACTAAAACGGT** |  | **2, 200 bp** |
|
| **mtDNA 9 fragment AlP-9** | **10,056-10,079 (*nad1*) 11,599-11,622 (*nad2*)** |  | **Forward: TGTTTATTGTTTATTTACCTTTTC Reverse: ACAAACAACTGAAAATAACACACA** |  | **1, 567 bp** |
|
| **mtDNA 10 fragment AlP-10** | **11,071-11,091 (*tRNA*-Lys) 12,376-12,398 (*cytb*)** |  | **Forward: AATGGGTTGTCACATCCTGGT Reverse: TCAAAATCTGAAAACCCAAAACC** |  | **1, 336 bp** |
|
| **mtDNA 11 fragment AlP-11** | **11,848-11,869 (*nad2*) 13,623-13,644 (*cox3*)** |  | **Forward: TAAGATTTTTGTTTTGAGGGAG Reverse: CACTAAGACCCTCCATAACAAT** |  | **1, 805 bp** |
|
| **mtDNA 12 fragment AlP-12** | **13,148-13,172 (*cytb*) 223-247 (*nad4*)** |  | **Forward: ATTTTGGTTCTTGTTGTTTTTGTTT Reverse: ACACACAAACAACAACCAAAACTTC** |  | **1, 374 bp** |
|
