# Supplementary material for: Complete Mitochondrial Genomes of Chimpanzee- and Gibbon-Derived Ascaris Isolated from a Zoological Garden in Southwest China
Source: PLoS One. 2013 Dec 17;8(12):e82795. doi: 10.1371/journal.pone.0082795 (PMC3866200; doi:10.1371/journal.pone.0082795)
Supplement: Table S2 — Annotation of the mitochondrial genomes of chimpanzee Ascaris (cA) and gibbon Ascaris (gA). aThe inferred lengths of the amino acid sequences of 12 protein-coding genes. bNegative numbers indicate the overlap of adjacent genes. Ini/Ter codons: initiation and termination codons; nt: nucleotide; aa: amino acid. (DOC) [file pone.0082795.s007.doc]

**Table S2.** Annotation of the mitochondrial genomes of chimpanzee *Ascaris* (cA) and gibbon *Ascaris* (gA).

| **Gene/region** | **Positions and nt sizes (bp)** | | **Ini/Ter codons and (aa** ^a^ **sequences)** | | **Anticodons** | **Intergenic  nt (bp)** **^b^** |
| --- | --- | --- | --- | --- | --- | --- |
|  | **cA** | **gA** | **cA** | **gA** | **cA/gA** | **cA/gA** |
| ***nad4*** | **1-1230 (1230)** | **1-1230 (1230)** | **TTG/TAA (409)** | **TTG/TAA (409)** |  | **0/0** |
| **Non-coding region (NCR)** | **1231-1346 (116)** | **1231-1347 (117)** |  |  |  | **0/0** |
| ***cox1*** | **1347-2924 (1578)** | **1348-2925 (1578)** | **ATT/TAG (525)** | **ATT/TAG (525)** |  | **0/0** |
| ***tRNA-*Cys (C)** | **2924-2979 (56)** | **2925-2980 (56)** |  |  | **GCA/GCA** | **-1/-1** |
| ***tRNA-*Met (M)** | **2984-3044 (61)** | **2985-3045 (61)** |  |  | **CAT/CAT** | **4/4** |
| ***tRNA-*Asp (D)** | **3045-3104 (60)** | **3046-3105 (60)** |  |  | **GTC/GTC** | **0/0** |
| ***tRNA-*Gly (G)** | **3109-3164 (56)** | **3110-3165 (56)** |  |  | **TCC/TCC** | **4/4** |
| ***cox2*** | **3165-3863 (699)** | **3166-3864 (699)** | **TTG/TAG (232)** | **TTG/TAG (232)** |  | **0/0** |
| ***tRNA-*His (H)** | **3865-3919 (55)** | **3866-3920 (55)** |  |  | **GTG/GTG** | **1/1** |
| ***rrn*L** | **3920-4879 (960)** | **3921-4881 (961)** |  |  |  | **0/0** |
| ***nad3*** | **4880-5215 (336)** | **4882-5217 (336)** | **TTG/TAG (111)** | **TTG/TAG (111)** |  | **0/0** |
| ***nad5*** | **5216-6800 (1585)** | **5218-6802 (1585)** | **ATT/T (528)** | **ATT/T (528)** |  | **0/0** |
| ***tRNA-*Ala (A)** | **6801-6856 (56)** | **6803-6858 (56)** |  |  | **TGC/TGC** | **0/0** |
| ***tRNA-*Pro (P)** | **6859-6914 (56)** | **6861-6916 (56)** |  |  | **TGG/TGG** | **2/2** |
| ***tRNA-*Val (V)** | **6915-6971 (57)** | **6917-6973 (57)** |  |  | **TAC/TAC** | **0/0** |
| ***nad6*** | **6972-7406 (435)** | **6974-7408 (435)** | **TTG/TAG (144)** | **TTG/TAG (144)** |  | **0/0** |
| ***nad4*L** | **7409-7642 (234)** | **7411-7644 (234)** | **ATT/TAG (77)** | **ATT/TAG (77)** |  | **2/2** |
| ***tRNA-*Trp (W)** | **7643-7699 (57)** | **7645-7701 (57)** |  |  | **TCATCA** | **0/0** |
| ***tRNA-*Glu (E)** | **7700-7758 (59)** | **7702-7760 (59)** |  |  | **TTC/TTC** | **0/0** |
| ***rrn*S** | **7759-8458 (700)** | **7761-8460 (700)** |  |  |  | **0/0** |
| ***tRNA-*SerUCN (S*''*)** | **8475-8528 (54)** | **8477-8530 (54)** |  |  | **TGA/TGA** | **16/16** |
| **AT-rich (AT)** | **8529-9410 (882)** | **8531-9416 (886)** |  |  |  | **0/0** |
| ***tRNA-*Asn (N)** | **9411-9467 (57)** | **9417-9473 (57)** |  |  | **GTT/GTT** | **0/0** |
| ***tRNA-*Tyr (Y)** | **9478-9535 (58)** | **9484-9541 (58)** |  |  | **GTA/GTA** | **10/10** |
| ***nad1*** | **9536-10408 (873)** | **9542-10414 (873)** | **TTG/TAG (290)** | **TTG/TAG (290)** |  | **0/0** |
| ***atp6*** | **10414-11013 (600)** | **10420-11019 (600)** | **ATT/TAG (199)** | **ATT/TAG (199)** |  | **5/5** |
| ***tRNA-*Lys (K)** | **11022-11083 (62)** | **11028-11089 (62)** |  |  | **TTT/TTT** | **8/8** |
| ***tRNA-*LeuUUR (L*''*)** | **11089-11143 (55)** | **11095-11149 (55)** |  |  | **TAA/TAA** | **5/5** |
| ***tRNA-*SerAGN (S*'*)** | **11144-11194 (51)** | **11150-11200 (51)** |  |  | **TCT/TCT** | **0/0** |
| ***nad*2** | **11195-12038 (844)** | **11201-12044 (844)** | **TTG/T (281)** | **TTG/T (281)** |  | **0/0** |
| ***tRNA-*Ile (I)** | **12039-12099 (61)** | **12045-12105 (61)** |  |  | **GAT/GAT** | **0/0** |
| ***tRNA-*Arg (R)** | **12100-12153 (54)** | **12106-12159 (54)** |  |  | **ACG/ACG** | **0/0** |
| ***tRNA-*Gln (Q)** | **12157-12211 (55)** | **12163-12217 (55)** |  |  | **TTG/TTG** | **3/3** |
| ***tRNA-*Phe (F)** | **12219-12277 (59)** | **12225-12283 (59)** |  |  | **GAA/GAA** | **7/7** |
| ***cytb*** | **12278-13375 (1098)** | **12284-13381 (1098)** | **ATT/TAA (365)** | **ATT/TAA (365)** |  | **0/0** |
| ***tRNA-*LeuCUN (L')** | **13390-13445 (56)** | **13396-13451 (56)** |  |  | **TAG/TAG** | **14/14** |
| ***cox3*** | **13446-14213 (768)** | **13452-14219 (768)** | **GTT/TAG (255)** | **GTT/TAG (255)** |  | **0/0** |
| ***tRNA-*Thr (T)** | **14214-14268 (55)** | **14220-14274 (55)** |  |  | **TGT/TGT** | **0/0** |

^a^The inferred lengths of the amino acid sequences of 12 protein-coding genes.

^b^Negative numbers indicate the overlap of adjacent genes.

Ini/Ter codons: initiation and termination codons; nt: nucleotide; aa: amino acid.
