# Supplementary material for: Complete Mitochondrial Genomes of Chimpanzee- and Gibbon-Derived Ascaris Isolated from a Zoological Garden in Southwest China
Source: PLoS One. 2013 Dec 17;8(12):e82795. doi: 10.1371/journal.pone.0082795 (PMC3866200; doi:10.1371/journal.pone.0082795)
Supplement: Table S3 — Size and nucleotide composition of different genomic regions in 11 ascaridoids reported within Ascaridida. aAll protein-coding genes were accounted for. bIn base pairs. (DOC) [file pone.0082795.s008.doc]

**Table S3.** Size and nucleotide composition of different genomic regions in 11 ascaridoids reported within Ascaridida.

| **Species** |  | **PCGs^a^** | |  | **rRNAs** | |  | **tRNAs** | |  | **NCR** | |  | **AT-region** | | **References** |
| --- | --- | --- | --- | --- | --- | --- | --- | --- | --- | --- | --- | --- | --- | --- | --- | --- |
|  |  | **Size^b^** | **AT%** |  | **Size^b^** | **AT%** |  | **Size^b^** | **AT%** |  | **Size^b^** | **AT%** |  | **Size^b^** | **AT%** |  |
| ***Ascaris* sp. (Chimpanzee)** |  | **10280** | **70.3** |  | **1660** | **74.3** |  | **1250** | **70.9** |  | **116** | **75.9** |  | **882** | **84.7** | **This study**  **This study**  **Kim et al. (2006)**  **Liu et al. (2012)**  **Park et al. (2011)** |
| ***Ascaris* sp. (Gibbon)** |  | **10280** | **70.3** |  | **1661** | **74.3** |  | **1250** | **71.0** |  | **117** | **76.1** |  | **886** | **84.6** |  |
| ***Anisakis simplex*** |  | **10274** | **69.5** |  | **1656** | **74.3** |  | **1208** | **72.4** |  | **122** | **84.4** |  | **515** | **87.2** |  |
| ***Ascaris lumbricoides*** |  | **10279** | **70.1** |  | **1663** | **74.7** |  | **1260** | **70.7** |  | **117** | **76.1** |  | **905** | **84.7** |  |
| ***Ascaris lumbricoides*** |  | **10280** | **70.3** |  | **1661** | **74.4** |  | **1250** | **71.1** |  | **117** | **76.1** |  | **893** | **84.2** |  |
| ***Ascaris suum*** |  | **10280** | **70.4** |  | **1659** | **74.3** |  | **1250** | **70.3** |  | **116** | **75.9** |  | **917** | **83.5** | **Liu et al. (2012)**  **Okimoto et al. (1992)**  **Xie et al. (2011)**  **Xie et al. (2011)**  **Xie et al. (2011)**  **Xie et al. (2011)**  **Lin et al. (2012)**  **Park et al. (2011)**  **Jex et al. (2008)**  **Li et al. (2008)**  **Li et al. (2008)**  **Li et al. (2008)** |
| ***Ascaris suum*** |  | **10397** | **70.5** |  | **1661** | **74.7** |  | **1252** | **71.0** |  | **117** | **76.1** |  | **886** | **84.6** |  |
| ***Baylisascaris ailuri*** |  | **10287** | **67.9** |  | **1657** | **69.5** |  | **1241** | **67.0** |  | **117** | **71.8** |  | **1282** | **82.0** |  |
| ***Baylisascaris procyonis*** |  | **10289** | **68.6** |  | **1664** | **72.0** |  | **1246** | **69.6** |  | **118** | **77.1** |  | **1375** | **82.8** |  |
| ***Baylisascaris schroederi*** |  | **10290** | **67.1** |  | **1657** | **69.8** |  | **1241** | **67.3** |  | **114** | **75.4** |  | **1406** | **78.9** |  |
| ***Baylisascaris transfuga*** |  | **10290** | **67.6** |  | **1658** | **69.7** |  | **1244** | **67.5** |  | **117** | **71.8** |  | **1516** | **82.3** |  |
| ***Contracaecum rudolphii* B** |  | **10281** | **69.0** |  | **1650** | **72.3** |  | **1256** | **70.6** |  | **115** | **78.3** |  | **588** | **89.1** |  |
| ***Cucullanus robustus*** |  | **10271** | **69.7** |  | **1635** | **76.8** |  | **1218** | **73.2** |  | **124** | **62.9** |  | **658** | **87.4** |  |
| ***Toxocara canis*** |  | **10294** | **67.3** |  | **1617** | **69.6** |  | **1222** | **69.3** |  | **115** | **75.6** |  | **828** | **78.1** |  |
| ***Toxocara canis*** |  | **10308** | **67.2** |  | **1655** | **69.8** |  | **1251** | **68.5** |  | **111** | **76.6** |  | **975** | **79.5** |  |
| ***Toxocara cati*** |  | **10284** | **68.8** |  | **1651** | **71.5** |  | **1248** | **70.2** |  | **116** | **74.1** |  | **711** | **81.3** |  |
| ***Toxocara malaysiensis*** |  | **10297** | **67.8** |  | **1651** | **68.5** |  | **1252** | **70.3** |  | **112** | **74.1** |  | **936** | **78.4** |  |

^a^All protein-coding genes were accounted for.

^b^In base pairs.
